# Genome-Wide Identification and Expression Analysis of Two-Component System Genes in Tomato

**DOI:** 10.3390/ijms17081204

**Published:** 2016-07-26

**Authors:** Yanjun He, Xue Liu, Lei Ye, Changtian Pan, Lifei Chen, Tao Zou, Gang Lu

**Affiliations:** 1 Department of Horticulture, Zhejiang University, Hangzhou 310058, China; hyj1009@163.com (Y.H.); 17816860626@163.com (X.L.); 707137378@qq.com (L.Y.); wpanchangtian@163.com (C.P.); 704909820@qq.com (L.C.); 1275051219@qq.com (T.Z.); 2 Key Laboratory of Horticultural Plant Growth, Development and Biotechnology, Agricultural Ministry of China, Hangzhou 310058, China

**Keywords:** tomato, two-component system, phylogeny, evolution, expression profiles

## Abstract

The two-component system (TCS), which comprises histidine kinases (HKs), phosphotransfers (HPs), and response regulator proteins (RRs), plays pivotal roles in regulating plant growth, development, and responses to biotic and abiotic stresses. TCS genes have been comprehensively identified and investigated in various crops but poorly characterized in tomato. In this work, a total of 65 TCS genes consisting of 20 HK(L)s, six HPs, and 39 RRs were identified from tomato genome. The classification, gene structures, conserved domains, chromosome distribution, phylogenetic relationship, gene duplication events, and subcellular localization of the TCS gene family were predicted and analyzed in detail. The amino acid sequences of tomato TCS family members, except those of type-B RRs, are highly conserved. The gene duplication events of the TCS family mainly occurred in the RR family. Furthermore, the expansion of RRs was attributed to both segment and tandem duplication. The subcellular localizations of the selected green fluorescent protein (GFP) fusion proteins exhibited a diverse subcellular targeting, thereby confirming their predicted divergent functionality. The majority of TCS family members showed distinct organ- or development-specific expression patterns. In addition, most of TCS genes were induced by abiotic stresses and exogenous phytohormones. The full elucidation of TCS elements will be helpful for comprehensive analysis of the molecular biology and physiological role of the TCS superfamily.

## 1. Introduction

A two-component system (TCS) via phosphorylation between histidine and aspartic-acid residues was first identified in bacteria [1,2]. The TCS system in bacteria consists of a membrane-associated histidine protein kinase (HK) and a cytoplasmic response regulator (RR) with a receiver (REC) domain. HK proteins sense environmental signals and autophosphorylate the histidine residue (H) of the HK domain; the phosphate is then transferred to an aspartate residue (D) of the REC domain of the RR protein [1,2]. A complex TCS signaling system has been identified in eukaryotic species, including higher plants [3]. Plant TCS components typically consist of three signal elements: hybrid HKs, histidine-containing phosphotransfers (HPs), and RRs [4,5]. *Arabidopsis* cytokinin signaling is a typical representative of TCS signal systems. Three transmembrane histidine kinases, namely, *AHK2*, *AHK3*, and *AHK4* function as cytokinin receptors and negatively respond to stresses in *Arabidopsis* cytokinin signaling [3,4,5,6]. These kinases perceive stimulus and are autophosphorylated at a conserved histidine residue in transmitter domain. The phosphory groups are then transferred to HPs at a conserved aspartate residue. Finally, HPs transmit the signal to receiver domain in type-B RRs, which could function as transcription factor to response to various environmental signals via numerous *cis*-elements in the promoter of type-A ARRs and further regulate the expression of downstream stress-related genes [3,4,5,6].

Take advantage of available whole genomic sequences, various TCS genes have been successfully identified and investigated in several plant species, including *Arabidopsis* [4], rice [7], maize [8], soybean [9], and Chinese cabbage [10]. Plant TCS elements play vital roles in responses to abiotic stress, particularly drought, high salinity, and high or low temperature. Most of TCS elements in *Arabidopsis* interact with ABA to participate in drought, salt, and low temperature stresses [11,12,13,14,15,16,17,18,19]. The expression level of *AHP1*, *AHP2*, and *AHP3* in *Arabidopsis* are significantly repressed by heat stress [20]. In rice, *OsAHP1/2* knockdown seedlings respond to salt and drought stresses in different patterns [21]. *OsHK3* participates in ABA-induced antioxidant defense [22]. In soybean, the expressions of most TCS genes are sensitive to dehydration [23]. The roles of some tomato TCS members involved in stress responses have been studied. The pollens of tomato *never-ripe* (*Nr*) mutant, a histidine kinase mutant, are more sensitive to heat stress via affecting pollen carbohydrate metabolism [24]. Some phytochromes (PHYs), function as histidine kinases and participate in response to drought stress [25].

Tomato, as a model of fleshy fruit plant, is an economically important fruit crop grown worldwide. The reproductive development of tomato is susceptible to various adverse environments, resulting in reduced yield and quality. TCS plays important roles in signal transduction involved in stress responses and plant development. The tomato ethylene receptor and PHY subfamily, which both belong to HK(L) family have been identified and investigated [26,27]. Some tomato ethylene receptor and PHY elements have been proved to play important roles in plant reproduction development. Transgenic plants with reduced ethylene receptor *LeETR4* expression levels enhance flower senescence, and affect fruit set [28]. Moreover, the PHY subfamily genes can modulate carotenoid levels and regulate the time required for phase transition during fruit ripening [29]. However, tomato TCS genes have not been systematically investigated. In this study, the putative TCS elements in tomato were identified through in silico analysis. The classification, chromosome distribution, and evolutionary relationships of the TCS gene family were predicted and analyzed. Subcellular localizations were predicted and verified based on the transformation of onion epidermal cells. The expression profiles of some identified TCS genes were determined using quantitative real-time PCR analysis (qRT-PCR) to assess their responses to different abiotic stresses and plant hormones. Our comprehensive analyses of the TCS elements in tomato may provide a framework for future studies to elucidate the function of the TCS family genes in stress tolerance and hormone response in tomato.

## 2. Results

### 2.1. Identification of the TCS Genes in Tomato

BLASTP searches were performed in Sol GenomiSl Network (http://solgenomiSl.net/) to explore putative TCS members in tomato by employing 280 TCS protein sequences from *Arabidopsis* [4], rice [7], maize [8], soybean [9], wheat [30], and Chinese cabbage [10] as queries. A total of 211 non-redundant sequences including 71 HK(L)s, eight HPs, and 132 RRs putative hits in tomato genome database were identified. The putative TCS proteins were searched with HMMER 3.0 by using the global HMM profile of the TCS characteristic domains. A total of 55, seven, and 56 non-redundant putative HK(L)s, HPs, and RRs were identified, respectively. The sequences obtained by above two methods were compared to remove redundancy. The non-redundant proteins were filtered further using Pfam and SMART based on the presence of structural characteristics and conserved domains of TCS elements. Finally, 65 TCS members consisting of 20 HK(L)s, six HPs, and 39 RRs were confirmed in tomato. All tomato TCS members were named according to the homologous genes in *Arabidopsis*. This nomenclature has also been used in soybean and Chinese cabbage [9,10]. TCS genes have been intensively studied in some model plant species and important crops. TCS gene family in tomato contains 65 members, which is bigger than that of all reported species except *Glycine max* (98)*, and Brassica rapa* (85) (Table 1).

### 2.2. HK Proteins in Tomato

Twenty HK(L) proteins were identified in tomato and categorized into nine HKs and 11 HK-likes (HKLs) based on the presence of the conserved His-kinase transmitter (HK) domain. Nine HK proteins were further classified into five subgroups: three cytokinin receptor-like SlHKs, three ethylene receptor-like SlHKs, one CKI1-like SlHK, one CKI2/AHK5-like SlHKs, and one AHK1-like SlHK. All of these HKs possess a conserved HK domain that contains five conserved signature motifs, namely, H, N, G1, F, and G2 [4]. Additionally, all 11 SlHKLs were divided into three subgroups: five PHY-like SlHKLs, two PDK-like SlHKLs, and other four ethylene receptor-like SlHKLs, in which the H sites of the HK domain is replaced by other amino acids (Table S1).

**Figure 1:**
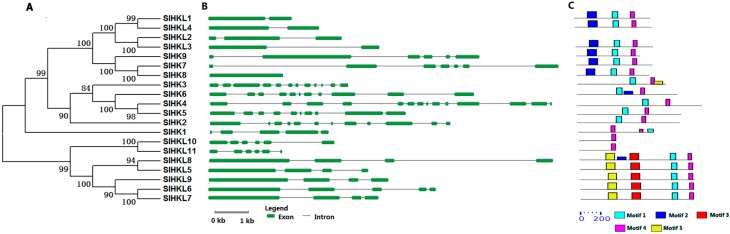
Phylogenetic analysis, gene structure, and conserved motifs of all HK(L) genes in tomato. (**A**) The phylogenetic tree of HK(L) proteins. Predicted amino acid sequences of HK(L) proteins were aligned using the Clustal X v1.81 program. The phylogenetic tree was constructed using the neighbor-joining (NJ) method with 1000 bootstrap replicates as implemented in the MEGA 5.0; (**B**) Gene structure was analyzed using the Gene Structure Display Server online. The green boxes indicate the exons, and lines indicate the introns; (**C**) Schematic distribution of conserved motifs in the HK(L) proteins. Motif analysis was performed using MEME 4.0 software as described in the methods. The colored boxes represent different motifs in the corresponding position of each HK(L) protein.

Three cytokinin receptor-like SlHKs, namely SlHK4, SlHK5, and SlHK6 exhibit conserved protein structure and high sequence identity (62%–64%) with *Arabidopsis* AHK4–AHK6. Gene structure analysis showed that *SlHK4*, *SlHK5*, and *SlHK6* possess 9–10 introns. These SlHK proteins contain two conserved motifs (motifs 1 and 4), as identified by MEME, as well as four conserved domains, namely, HK, REC, CHASE, and transmembrane (TM) domains, as recognized by Pfam and SMART online tools (Figure 1 and Figure S1). Multiple sequence alignment showed that CHASE domains are highly conserved among tomato cytokinin receptors (Figure S2), and CHASE domain is crucial for proteins to recognize and bind cytokinin [4]. Tomato SlHK1 has 38% identity with CKI1 in *Arabidopsis* and *CKI1*, which is involved in cytokinin signaling and development of female gametophytes in *Arabidopsis* [5].

Tomato ethylene receptors and PHY members were identified in previous study [26,27]. Tomato ethylene receptors contain seven members (SlHK7-SlHK9 and SlHKL1-SlHKL4), which contain a C2H2-type zinc-finger (C2H2) domain as an ethylene-binding domain (Table S1). Among these genes, *SlHKL1*-*SlHKL4* contain one or two introns, similar exon-intron architecture were also found in *Arabidopsis* homologous genes. However, *SlHK7* and *SlHK9* have five or six introns (Figure 1, Table S1). It should be noted that there is no intron in *SlHK8* gene, although it has 90% amino sequence with SlHK7 (Table S2). In addition, we identified a new ethylene receptor EIN4-like gene, namely *SlHKL2*, which shows 59% sequence identity with *Arabidopsis* EIN4. SlHKL2 contains characteristic domains, namely, TM, GAF, HKL, and REC. Tomato PHY genes were previously named *PHYA*, *PHYB1*, *PHYB2*, *PHYE*, and *PHYF* [27]. The tomato PHYF is a homolog of *Arabidopsis* PHYC. In the present study, these genes which were renamed as *SlHKL5–9*, show 59% to 78% sequence similarity to their counterparts in *Arabidopsis* and all genes contain GAF, PHY, PAS, and HKL domains (Figure S2). The PHY members in *Arabidopsis* function as red or far-red light photoreceptors and participate in various photomorphogenic processes [33].

### 2.3. HP Proteins in Tomato

**Figure 2:**
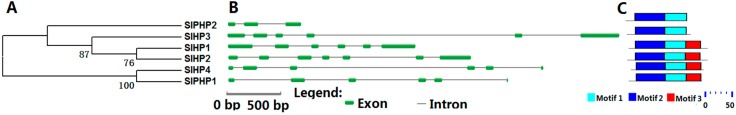
Phylogenetic analysis (**A**); gene structure (**B**); and conserved motifs (**C**) of the HP family members in tomato. For other details, see Figure 1.

We identified six HP proteins in tomato with four authentic and two pseudo-HPs. Each of the six SlHP genes contains five or six introns except *SlPHP2*. All SlHP proteins exhibit 53% to 70% identity to their homologs, namely, AHP1, AHP4, and APHP1 in *Arabidopsis*. All tomato HPs contain two conserved motifs (motifs 1 and 2) (Figure 2, Table S1). HP proteins generally possess the Hpt domain with a signature motif of XHQXKGSSXS. However, in SlPHP1 and SlPHP2, the His of Hpt domain is replaced by Tyr and Asn, respectively (Figure S3). Although SlPHP1 lost the conserved Hpt domain, it shows a high identity (63%) to authentic HP (AHP4) in *Arabidopsis*.

### 2.4. RR Proteins in Tomato

**Figure 3:**
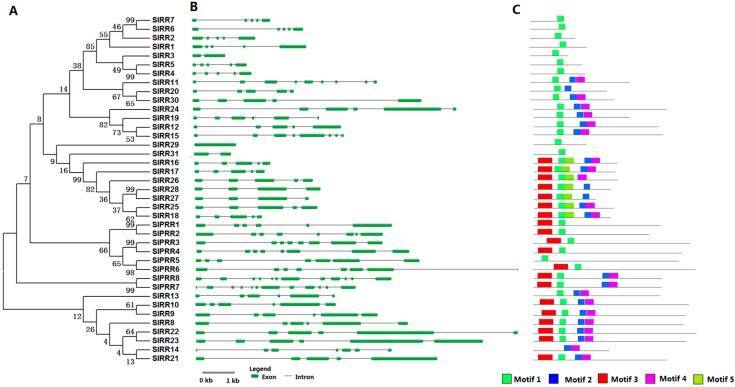
Phylogenetic analysis (**A**); gene structure (**B**); and conserved motifs (**C**) of RR genes in tomato. For other details, see Figure 1.

Thirty-nine RRs, including seven type-A RRs, 23 type-B RRs, one type-C RR, and eight pseudo-RRs (PRRs) were identified in tomato (Table S1). Tomato type-A RRs, including SlRR1–7, have much smaller average protein size compared with these of other RRs and share a high degree sequence identities (58% to 79%) with their counterparts (ARR3, ARR9, and ARR17) in *Arabidopsis*. They exhibit quite similar gene structure. All tomato type-A RRs possess four introns, except for *SlRR3*, which contains only one intron (Figure 3). These type-A RRs only contain one conserved REC domain and only correspond to motif 3, as identified by MEME (Figure 3 and Figure S4). Plant type-B RR proteins are usually featured by REC domain in N-end and Myb domain in C-end. However, nearly half of tomato type-B RRs only contain a REC domain, where the Myb domain may be lost during the evolution of the tomato RR family. Notably, except REC domain, SlRR29 protein also has a Trans-reg-C domain, which is only present in prokaryote TCS element (Figure S5). This finding indicates that eukaryote TCS elements are probably evolved from that of prokaryotes. Only one type-C RR, SlRR31, was identified in tomato; this protein shares 37% identity with *Arabidopsis* ARR22 and contains only one REC domain which is similar to the structure of type-A RR proteins. Tomato PRR subfamily was further classified into six clock and two type-B PRRs (Table S1). *SlPRR1*–*6* belonging to clock PRRs, share 37% to 68% sequence identity with the homologous proteins, namely, APRR1, APRR5, and APRR7, in *Arabidopsis*. All six clock PRRs have two motifs (motifs 1 and 3), except SlPRR5, which lose its pseudo-REC domain. SlPRR1–6 contain a CCT domain, which plays an important role in regulating circadian rhythm and controlling flowering time [34]. Only two type-B PRRs, namely, SlPRR7 and SlPRR8, were identified to be homologous with APRR2 in *Arabidopsis* with 45% and 46% sequence identities, respectively. SlPRR7 and SlPRR8 are characterized by a pseudo-REC and a Myb domain (Figure 3). *SlPRR8* regulates tomato plastid development and fruit ripening [35].

### 2.5. Phylogenetic Analysis of Plant TCS Proteins

**Figure 4:**
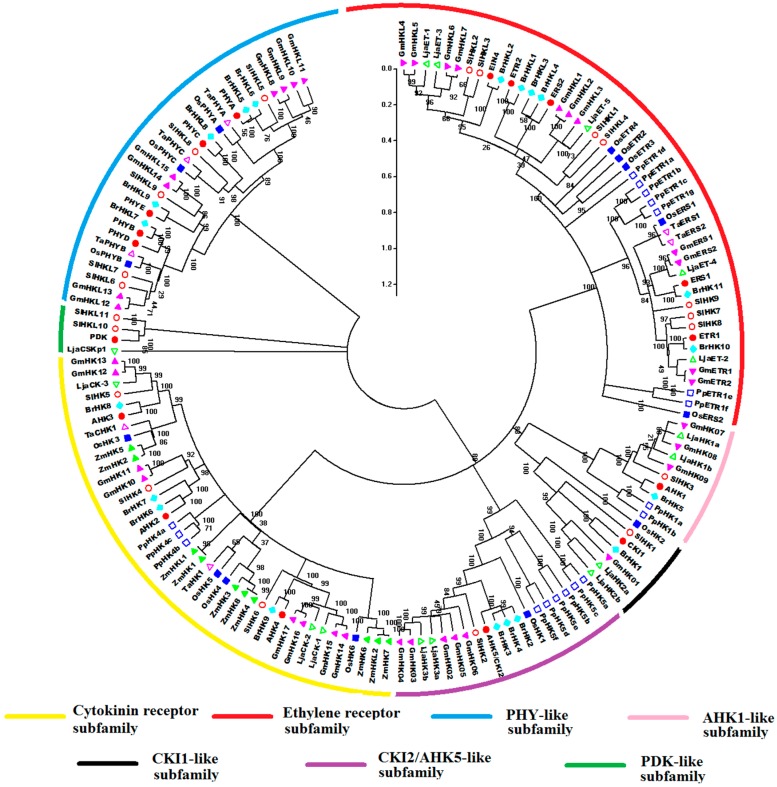
Phylogenetic relationship of HK(L) proteins in *Arabidopsis*, rice, maize, Chinese cabbage, soybean, *Lotus japonicus*, *Physcomitrella patens*, wheat, and tomato. The phylogenetic trees were constructed using the NJ method with bootstrap 1000 tests by MEGA 5.0. The diverse subgroups of HK(L) proteins were marked by different colors. The bar represents the relative divergence of the sequences examined.

All amino acid sequences of HK(L) proteins from *Arabidopsis*, rice, maize, soybean, Chinese cabbage, *Lotus japonicus*, *Physcomitrella patens*, wheat, and tomato were used to perform multiple alignments and generate phylogenetic trees for exploring the evolutionary relationships of these HK(L)s (Figure 4). The HK(L) proteins in these nine species were divided into seven distinct clades, the cytokinin receptor, ethylene receptor, PHY-like, CKI1-like, CKI2/AHK5-like, AHK1-like, and PDK-like subfamilies; this finding is similar to that reported in previous studies [5,10]. Tomato HL(L)s usually have much closer relationships to that of soybean and *Lotus japonicas* than that of other species, which both are leguminous crops. As expected, the members from *Physcomitrella patens*, the only moss in the nine species, are generally distinct from the members of other species in each subclade. Unlike the other subfamilies, ethylene receptor and PHY-like subfamily members show an alternating distribution of monocots and eudicots in the phylogenetic tree, hence, these two subfamilies likely occurred before the divergence of dicots and monocots.

**Figure 5:**
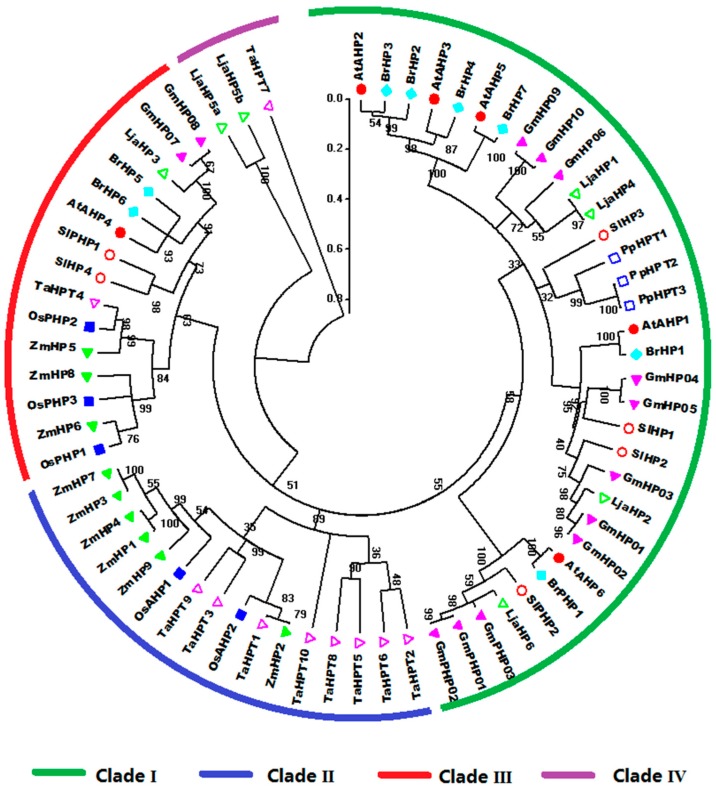
Phylogenetic phylogenetic analysis of plant HP family genes in *Arabidopsis*, rice, maize, Chinese cabbage, soybean, *Lotus japonicus*, *Physcomitrella patens*, wheat, and tomato. For other details, see Figure 4.

The phylogenetic tree divides HP proteins from the nine species into four clades, namely, I, II, III, and IV (Figure 5). Clade I is only occupied by HPs from dicots. Notably, the HPs from *Physcomitrella patens* are grouped into clade I and have closest relationship with SlHP3 in tomato. Meanwhile, the HPs in clade II are all from monocots. Clade III is further divided into two subclades consisting of the HP members from monocot and dicot, respectively.

**Figure 6:**
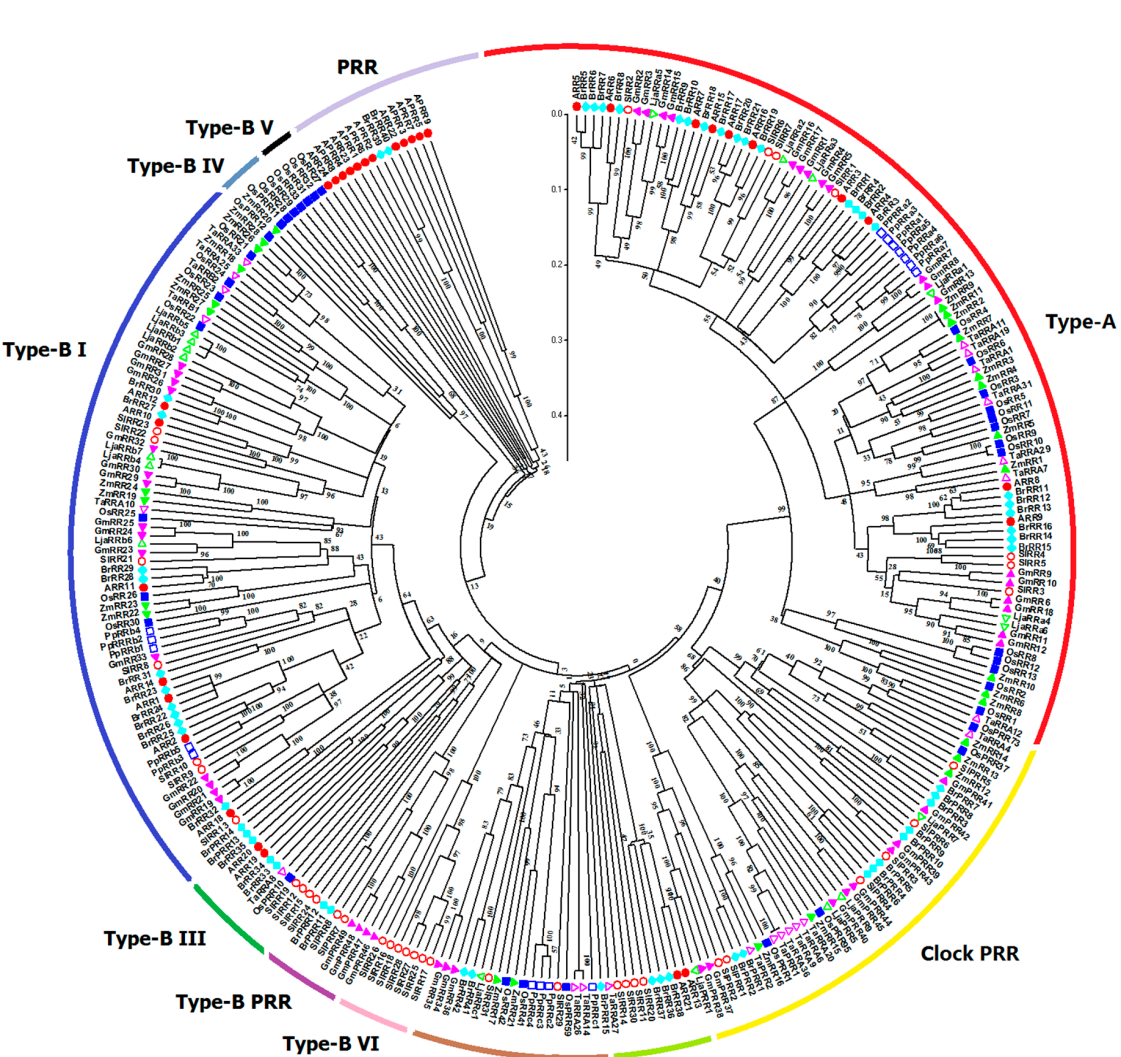
Phylogenetic relationship of RR proteins in *Arabidopsis*, rice, maize, Chinese cabbage, soybean, *Lotus japonicus*, *Physcomitrella patens*, wheat, and tomato. For other details, see Figure 4.

All the RR proteins from the nine species were grouped into type-A, type-B, type-C, and PRR subfamily (Figure 6). Generally, tomato RRs are phylogenetically closer to soybean and *Lotus japonicus* RRs. Phylogenetic analyses showed that type-A RRs from these nine species share a fairly close evolutionary relationship to each other. Type-B RRs, the biggest subgroup, could be further divided into six subgroups. Type-B I RRs are the most predominant RRs and contain the RRs from the nine species. Type-B IV and V subgroups only contain RRs from monocots. And type-B VI subgroup is exclusively occupied by tomato RRs (SlRR16–18 and SlRR25–28), as a tomato-special subgroup. Previous studies suggested that type-C RRs may be the oldest RR genes; type-A RR, as the youngest subgroup, may evolved from type-C RRs by mutations happened in their promoter sequences [36]. Type-C RRs share similar structure, whereas have lower similarity with type-A RR proteins. All monocot and eudicot type-C RRs show an alternating distribution in the phylogenetic tree; this finding indicates that these type-C RRs probably already existed before the divergence of monocotyledons and dicotyledons. The type-C RRs from *Physcomitrella patens* exhibit closer relationship with RRs from monocotyledons. Notably, *Arabidopsis* APRRs are clustered into a distinct subclade in the phylogenetic tree, whereas PRRs from the other species could be further divided into clock and type-B PRR subclades. The type-B PRRs are closely related to type-B RRs, which is consistent with the results of previous phylogenetic analyses [9,10].

### 2.6. Genomic Distribution and Gene Duplication of Tomato TCS Members

**Figure 7:**
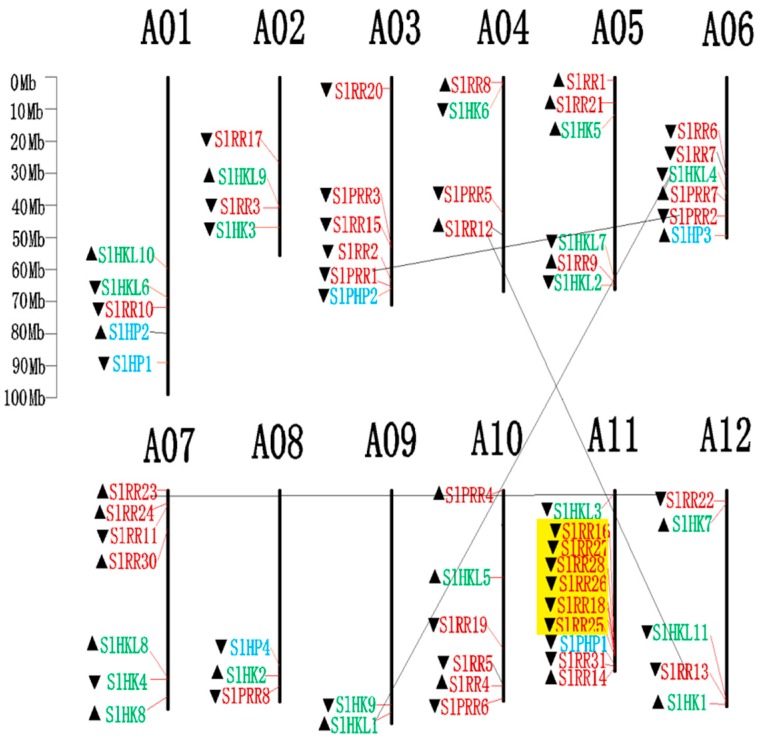
Chromosomal distribution of TCS genes in tomato. The chromosome number is indicated at the top of each chromosome. The arrows indicate the sense (▲) and antisense (▼) strands. The pairs of genes with tandem duplication were highlighted with the yellow background. The duplicated gene pairs have been link by dark line.

All identified tomato TCS family genes, except *SlRR29,* located on the scaffcold A00, are distributed on 12 tomato chromosomes (Figure 7). The HK(L)s are unevenly mapped on all tomato chromosomes except A03. Six SlHPs are located on tomato chromosome A01, A03, A06, A08, and A11, whereas 39 SlRRs are mapped on all the tomato chromosomes, except chromosome A09.

The gene duplication events were analyzed in the tomato TCS gene family. The duplicate pairs result from segment duplication, including *SlHKL1*/*SlHKL4*, *SlRR12*/*SlRR13*, *SlRR22*/*SlRR23*, and *SlPRR1*/*SlPRR2*, respectively. On the other hand, the duplication genes of *SlRR16*, *SlRR18*, and *SlRR25–SlRR28*, which are clustered on chromosome A11, were identified as tandem duplicates. All these results suggest that segmental and tandem duplication probably contribute to the expansion of the tomato TCS gene, which differs from that in *Arabidopsis*, Chinese cabbage, and soybean [5,9,10].

The synonymous rate (*Ks*), non-synonymous rate (*Ka*), and *Ka*/*Ks* of these duplicates were calculated, and duplication time was speculated using the values of *Ks* (Table 2). The *Ks* of four segment duplicates range from 0.6 to 0.79. Thus, the divergent time ranges from 46.15 Mya to 60.77 Mya. The *Ka/Ks* values of the segment duplicates are less than 1, indicating that they underwent purify selection. Meanwhile, the tandem cluster of *SlRR16*, *SlRR18*, and *SlRR25*-*SlRR28* were speculated to diverge from 5.97 Mya to 26.55 Mya. The *Ka/Ks* values of all these duplicates are less than 1, indicating that that purification selection occurred in these duplicates except in the duplicated pair of *SlRR18/26*.

### 2.7. Analysis of Cis-Elements in Putative Promoter Regions of TCS Genes in Tomato

We identified and analyzed cis-regulatory elements in the putative promoter regions of the TCS genes in tomato (Table S3). Numerous cis-motifs are involved in responses to abiotic stresses (high or low temperature, wound, and drought) and hormone treatments (ethylene, MeJA, salicylic acid, and ABA). ABA (ABRE and CE3) and drought (MBS) related cis-elements were detected in 21 out of total 65 gene promoters. Only seven TCS members contain the low temperature-responsive elements (LTR). Interestingly, we identified 38 heat stress-responsive elements (HSE) in the putative promoter regions of tomato TCS genes. Consistently, it has been reported that the pollens of the *SlHK9* mutant were more sensitive to heat stress [24].

### 2.8. Subcellular Localization of TCS Proteins from Tomato

**Figure 8:**
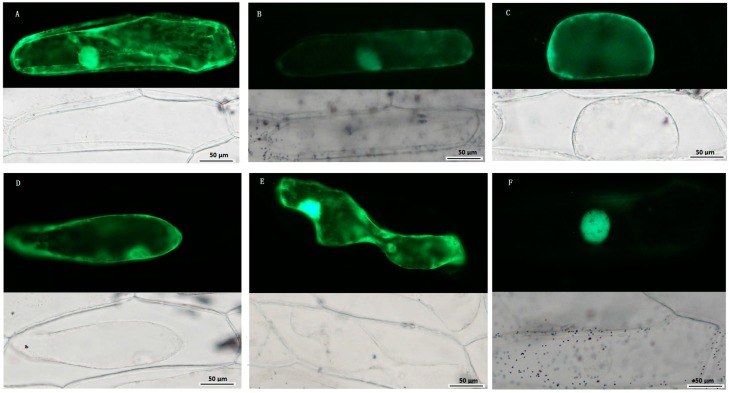
Subcellular localization of TCS proteins. Green fluorescent protein (GFP)-fusion proteins were transiently expressed in onion epidermis cells under the control of the 35S promoter. After 16–18 h of incubation, GFP signal was detected with a green fluorescence microscope. Fluorescence (**up**) and bright-field images (**down**) of plasmolyzed empty vector pFGC: GFP transgenic cell (**A**); Fluorescence (**up**) and bright-field images (**down**) of 35S::SlHK8-GFP (**B**); 35S::SlHP2-GFP (**C**); 35S::SlHP3-GFP (**D**); 35S::SlRR1-GFP (**E**); and 35S::SlRR8-GFP (**F**) transgenic cell. Scale bar was presented in bottom right.

Subcellular localization of some TCS proteins was analyzed using transiently expression via green fluorescent protein (GFP)-fusion proteins in the epidermal cells of onion. SlHK8, as an ethylene receptor, was predicted to be located in cytoplasm using SubLoc v1.0 website [37] (Table S1). But, in fact, besides cytoplasm, it was also detected to be located in nuclear, membrane, and cell walls, indicating that SlHK8 probably serves as membrane bound receptor. SlHP2 and SlHP3 were predicted to be located in cytoplasm and mitochondrion, respectively. Consistently, the fluorescence signal of SlHP2-GFP and SlHP3-GFP proteins was mainly detected in cytoplasm and membrane. It is worth noting that SlHP3-GFP fluorescence signal was also detected in the nucleus. SlRR8 is located in the nucleus, which is consistent with its function as a transcription factor. As a type-A RR, the fluorescence signal of the SlRR1-GFP protein was clearly detected in the nucleus, whereas a weak signal was also found in the cytoplasm (Figure 8). Similarly, all of the RR proteins in *Arabidopsis* were found to be localized exclusively in the nucleus except type-A ARRs, namely, ARR3 and ARR16, which are also localized to the cytoplasm [38]. Thus, the subcellular localizations of the five selected tomato TCS proteins are highly similar to that of the homologous proteins in *Arabidopsis* and display a diverse subcellular targeting, indicating their predicted divergent functionality.

### 2.9. Expression Profiles of Tomato TCS Genes in Various Organs

**Figure 9:**
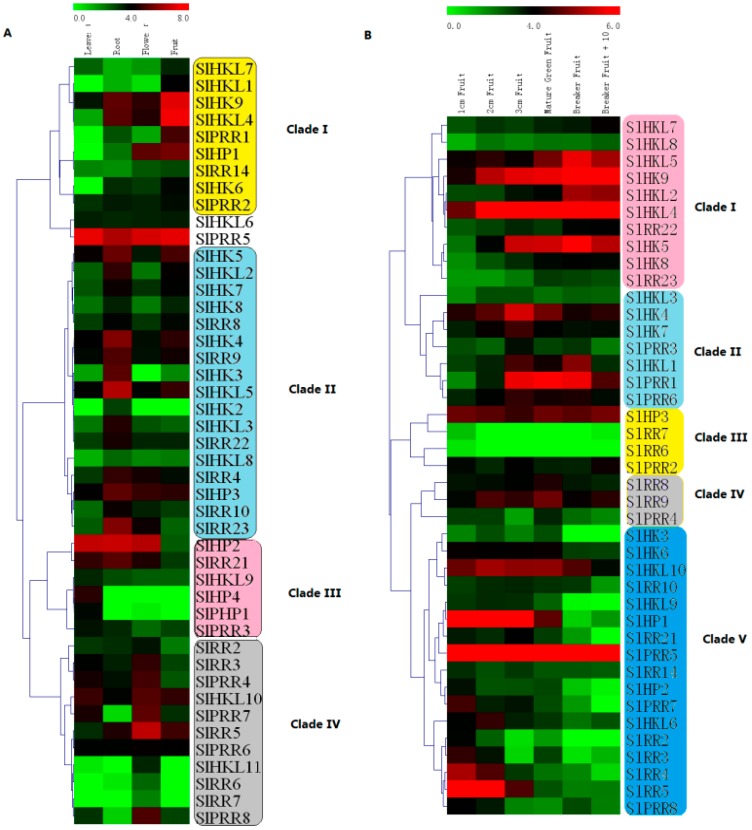
Heat map representation for organ-specific expression (**A**) and six fruit development stages-related expression (**B**) profiles of TCS genes in tomato. These electronic expression data were downloaded from the tomato eFP browser at bar.utoronto.ca. The heatmap was drawn by MeV4.8. The expression levels are presented using fold-change values transformed to Log_2_ format compared with control. The Log_2_ (fold-change values) and the color scale are shown at the top of heat map. Green, black, and red represent low, medium, and strong expression, respectively.

The electronic expression profiles of 65 tomato TCS genes in various organs/tissues were downloaded from the tomato eFP browser at bar.utoronto.ca. Among them, the transcripts of 20 TCS genes were quite low in all detected organs/tissues. So we clustered the rest 45 gene expression profiles in various organs with MeV4.8. The heatmap indicated that the expression profiles of tomato TCS genes in leaf, root, flower, and fruit could be divided into four clades (Figure 9A). The genes in clade I highly expressed in tomato fruit, whereas most of them had a quite low expression level in leaf. In clade II, highest mRNA levels of 17 genes were detected in root. The genes from clade III are predominantly expressed in leaf. Notably, the transcripts of *SlHP4* and *SlPHP1*, along with their homologous gene *AHP4* in *Arabidopsis* [4], were specifically detected in the leaves. There were 11 genes were found express highest in flower and grouped into clade IV. Among them, the transcripts of *SlHKL1*, *SlRR6/7*, and *SlPRR8* were only detected in flower.

The expression profiles of tomato TCS genes in six fruit different developmental stage were further summarized and clustered (Figure 9B). They could be further grouped into five clades (Figure 9B). In clade I, the gene expression levels gradually increase during fruit development. Clade II members are expressed highly at the middle stage of fruit development. In contrast with that in clade I, the gene expression levels in clade V exhibit a decrease trend during fruit development with the lowest level at 10 d after breaker. As expected, all of type-A RRs, except SlRR6 and SlRR7, are clustered in clade V and exhibit a high expression level in early fruit, indicating that they may be involved in fruit development.

**Figure 10:**
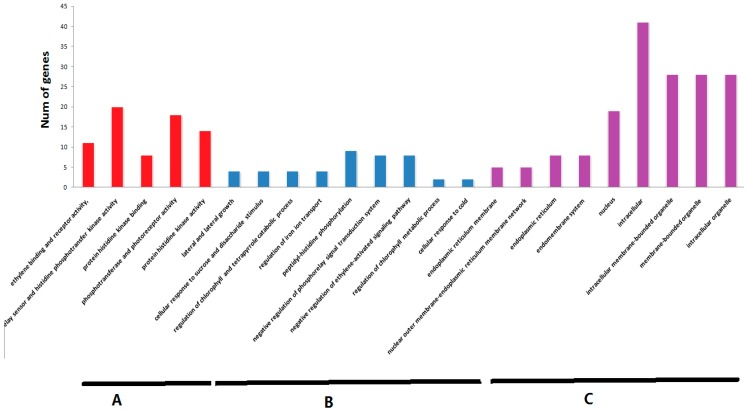
The Gene Ontology (GO) analysis of TCS genes. The TCS genes were categorized into three groups: molecular function (**A**); biological process (**B**); and cell component (**C**).

The selected 45 tomato TCS genes were subjected to Gene Ontology (GO) enrichment analysis (Figure 10). GO analysis indicated that TCS genes are mainly associated with three molecular functions, namely, ethylene binding and receptor activity, kinases binding and activity, phosphotransfer and photoreceptor activity. Coordinately, these genes are mainly located in nucleus, intracellular, reticulum and endomembrane system, and intrcellar membrane-bounded organelle. However, they are involved in many biological processes, such as regulation of peptidyl-histidine phosphorylation, negative regulation of phosphorelay signal transduction system and ethylene-activated signaling pathway, lateral and secondary growth, cellular response to sucrose stimulus, regulation of chlorophyll and tetrapyrrole catabolic process, regulation of iron ion transport, and cellular response to cold. In additional, KEGG pathways that were significantly enriched in the TCS genes were shown in Table S4. As expected, the mainly enriched pathway mapped in these tomato TCS genes include plant hormone signal transduction (ko: 04075), circadian rhythm (ko: 04712), and two-component system (ko: 02020), which is consistent with the findings of function studies in *Arabidopsis* TCS [4].

### 2.10. Expression Profiles of TCS Genes in Response to Exogenous Hormones and Abiotic Stresses

**Figure 11:**
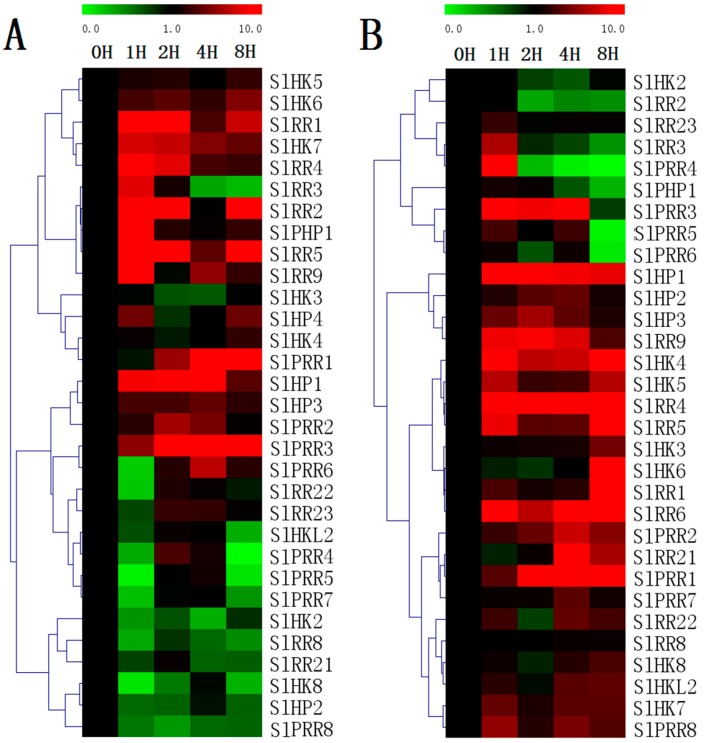
Heat map representation for the response patterns to exogenous trans-zeatin (ZT) (**A**) and ABA (**B**) of TCS genes in tomato. The second true leaves were collected at 0, 1, 2, 4, and 8 h after 100 μM ZT or 100 μM ABA treatment. The heatmap were manufactured by MeV4.8. The color scale representing the relative expression values is shown in the upper left of the heatmap.

Evaluation of the expression levels of 31 randomly selected TCS genes revealed that most of the detected TCS genes in tomato could be induced by exposure to exogenous trans-zeatin and ABA treatment (Figure 11). However, the expression patterns vary among distinct TCS genes. For ZT treatment, all the detected tomato type-A RRs, namely, *SlRR1*–*SlRR5*, are generally induced by ZT, particularly at 1 h after treatment. Similarly, the type-A RRs in other species were proven to be obviously upregulated by cytokinin [5,10]. All the detected tomato SlHKs display different response profiles to ZT treatment. *SlHK8* are obviously downregulated. However, *SlHK6–7* could be induced by ZT. Almost all of the detected SlHPs are generally induced, except *SlHP2*, which is obviously downregulated. Under ABA treatment, tomato HK transcripts are generally induced and their expression levels are maintained at a relatively high level at 8 h. By contrast, most of HPs are induced at the early stages but then decrease to a low level at 8 h except *SlHP1*. In addition, most of SlRRs positively respond to ABA except *SlRR2–3*, *SlRR23*, and *SlPRR4–6*.

**Figure 12:**
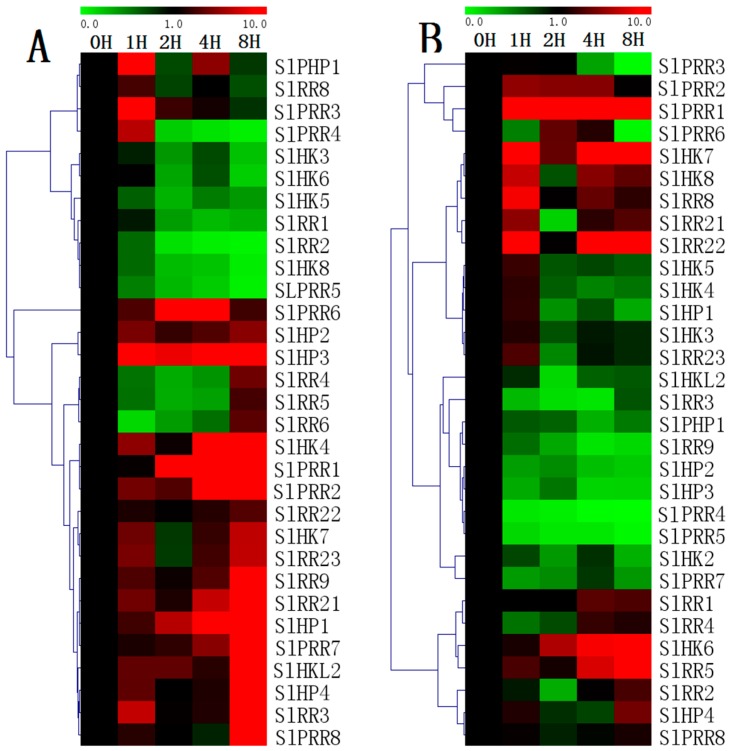
Heat map representation for the response patterns to drought (**A**) and salt (**B**) stresses of TCS genes in tomato. The second true leaves were collected at 0, 1, 2, 4, and 8 h after the onset of stress treatments. For other details, see Figure 10.

Almost all of the detected SlHPs are upregulated in response to drought stress. All of HPs are evidently induced from 1 h after drought treatment and generally maintain at a higher level, except that the transcript levels of *SlPHP1* decrease 2 h after treatment. However, all the tested SlHKs except *SlHK4/7* are downregulated after drought treatment. Except *SlRR3*, the expression of the other tomato type-A RRs are generally downregulated even though the transcript levels of *SlRR4–6* increase at 8 h. Except *SlPRR4–*5, all SlPRRs are obviously upregualted by drought. For salt treatment (Figure 12), most of HPs, including *SlHP1*, *SlHP2*, *SlHP3*, and *SlPHP1* are evidently downregulated in general after salt treatment, although *SlHP1* is slightly induced at 1 h. Similarly, a majority of SlHKs including *SlHK2*, *SlHK3*, *SlHK4*, and *SlHK5* are generally downregulated by salt stress, although S1HKs slightly decrease at 1 h. Notably, two ethylene receptors (*SlHK7* and *SlHK8*) are significantly induced by salt stress but repressed at 2 h. The other ethylene receptor like gene, *SlHKL2*, is obviously repressed by drought. Interestingly, most TCS genes such as *SlHP4*, *SlPRR1*, *SlPRR2*, *SlPRR4*, and *SlPRR5* exhibit similar response patterns to drought and salt*.* However, some genes including *SlHK8*, *SlHP3*, *SlHP4*, and *SlRR23* exhibit an opposite expression patterns under two abiotic stresses.

## 3. Discussion

In this study, a total of 65 TCS genes, including 20 HK(L)s, six HPs and 39 RRs, were identified from tomato genome. The number of TCS family members in tomato is slightly bigger than that of *Arabidopsis* (56), rice (52), and maize (59), but obviously fewer than that in *Glycine max* (98), and *Brassica rapa* (85) (Table 1). In detail, the number of HK(L) family members in tomato (20) is larger than that in *Arabidopsis* (17), rice (11), and maize (11), which is only less than that in soybean (36). It was worth mentioning that tomato contains the largest number of type-B RRs (23) in all identified species and is nearly twice as many as that in *Arabidopsis* (12). In tomato, four pairs of segment duplicates including *SlHKL1* and *SlHKL4*, *SlRR12* and *SlRR13*, *SlRR22* and *SlRR23*, and *SlPRR1* and *SlPRR2* were found. And a tandem duplicate cluster of *SlRR16*, *SlRR18*, and *SlRR25–SlRR28* were identified. These tandem duplicates with high similarties exhibit conserved protein and gene structure, which all have a REC domain. Furthermore they occupied a tomato-specific type-B VI subfamily in the phylogenetic tree. Segmental duplication and tandem duplication events both contribute to the expansion of the TCS gene family in tomato. By contrast, in *Arabidopsis*, Chinese cabbage, and soybean, segmental duplication was the main mechanism contributing to the duplication of TCS genes [5,9,10]. In *Arabidopsis*, 10 pairs of segmental duplicates were found which accounted for 35.71% of all *Arabidopsis* TCS genes [5,10]. In Chinese cabbage, 61 genes among all 85 TCSs were identified to be duplicated because of segmental duplication [10]. A total of 66 out of 98 soybean TCS genes were identified to be segment duplicates [9]. Tandem duplication was not found in the TCS genes from *Arabidopsis* and soybean. Only one pair of duplicated genes was identified in Chinese cabbage. These results suggested that, unlike that in tomato, segmental duplication might be the main mechanism contributing to the duplication of TCS genes in *Arabidopsis*, Chinese cabbage, and soybean [5,9,10]. In tomato TCS genes, all of the tandem duplication and half of segmental duplication occur in type-B RRs. Thus, the gene duplication of type-B RRs mainly contributes to the expansion of TCS. Previous studies demonstrated that tomato genome underwent two independent large-scale genome and/or segmental duplication events. One of these duplications was ancient and occurred around 170–235 Mya, immediately after the divergence of monocots and dicots. The other duplication was recent polyploidy duplication, which occurred approximately 90 Mya and is the estimated divergence time of tomato and *Arabidopsis* [39,40]. In this study, the *Ks* of segmental duplicates in the tomato TCS genes ranged from 0.6 to 0.79, which corresponded to the divergence time of 46 Mya to 60 Mya, suggesting that the gene duplication events occurred after the split of tomato and *Arabidopsis*. On the other hand, *SlRR16*, *SlRR18*, and *SlRR25*–*28* in the tomato genome clustered together on chromosome A11, forming a tandem duplicate cluster. The calculated divergence time was varied from 5.97 Mya to 26.55 Mya. Tandem duplicates usually occurred more recently than segment duplicates, which probably occurred because tandem duplications in plants were more likely to participate in stress responses and these tandem duplicates were not retained as long as nontandem duplicates [41] (Table 2).

Expression analysis indicated that 45 TCS genes in tomato are predominantly expressed in the root, fruit, or flower, whereas the transcripts of other genes could not be detected in any tomato organs (Figure 9). Twelve genes including *SlHK2–4*, *SlHKL3/5*, *SlHP2/3*, *SlRR9/10*, and *SlRR21–23*, are predominantly expressed in the roots where the cytokinins are mainly synthesized. These genes probably play important roles in cytokinin signal transduction like their homologous genes in *Arabidopsis* [5]. This result is consistent with the finding in Chinese cabbage and soybean [10,23]. Most of TCS elements including HKLs and PRRs, exhibit preferential expression in tomato fruit. Notably, all ethylene receptors, except *SlHKL3*, are highly expressed during fruit ripening stage. Ethylene receptors SlHK7–9 and SlHKL1–4 all have a C2H2 domain, which could perceive ethylene signal. In fact, the function of some TCS elements in the development and ripening of tomato fruit has been widely studied [28,29,35]. Transgenic plants with reduced *LeETR4* (*SlHKL4*) enhanced flower senescence and failed to fruit set [28]. Analysis on phytochrome *phyA, phyB1,* and *phyB2* single, double, or triple mutants indicated that they participated in modulating the carotenoid formation and the time required for phase transitions during fruit ripening [29]. Additionally, tomato *SlPRR8* was verified to regulate plastid development and fruit ripening [35].

Increasing evidence verified that TCS proteins are involved in responses to various abiotic stresses [10,17,18,19,23]. In this study, 31 tomato TCS genes were detected, and most genes appear to be regulated by drought- and salt-stresses (Figure 12). Most of tomato TCS genes negatively respond to salt stress, and similar results were found in soybean and Chinese cabbage [10,23]. However, 18 out of 31 genes are obviously induced by drought treatment, which differ from that in *Arabidopsis*, soybean and Chinese cabbage [10,17,18,19,23]. For examples, most of HPs and PRRs in tomato are upregulated by drought stress, but AHP and APRRs in *Arabidopsis* negatively responded to drought [18,19]. Similarly, tomato type-B RRs *SlRR9*, *SlRR22*, and *SlRR23* are upregulated by drought but their homologous genes *ARR1* and *ARR12* negatively responded to drought [17]. In additional, we identified 23 out of total 65 genes containing dehydration-inducible ABRE, CE3, and/or MBS motifs in their promoter regions (Table S3). The expression profiles of 12 out of all these 23 genes including *SlHK4/5/7*, *SlHP1*, *SlRR1/21/22*, and *SlPRR1/3/4/5/8* were analyzed by qRT-PCR. In detail, nine genes including *SlHK4/7*, *SlHP1*, *SlRR21/22*, and *SlPRR1/3/8* are generally induced by drought, which are consistent with the promoter analyses, but the other three genes (*SlHK5*, *SlRR1*, and *SlPRR5*) are downregulated. The inconsistent results of *SlHK5*, *SlRR1*, and *SlPRR5* between expression profiles and promoter analyses were also observed in soybean and Chinese cabbage [10,23]. Plant TCS elements were determined to play vital roles in responses to abiotic stresses, particularly drought, high salinity, and high or low temperature in *Arabidopsis,* rice, tomato, and soybean [11,12,13,14,15,16,17,18,19,20,21,22,23,24,25,42,43]. Tomato *LE-ETR3* (*Nr*) participated in salt and heat stresses, and reducing expression of *LE-ETR4* led to an enhanced hypersensitive response [24,42,43]. Meanwhile, tomato *PHYA, PHYB1*, and *PHYB2* were verified to modulate drought stress responses [25]. The expression analyses here for TCS elements in this work provide an important implication on the function of these family genes under abiotic stresses.

## 4. Materials and Methods

### 4.1. Identification of TCS Genes in Tomato

Protein sequences of all known plant TCS genes, particularly 56, 52, 51, 98, 62, and 85 members in the genome of *Arabidopsis*, rice, maize, soybean, *Lotus japonicus*, *Physcomitrella patens*, wheat, and Chinese cabbage, respectively, were downloaded from Phytozome [44] and then used as queries to perform BLASTP searches in the SGN database (http://solgenomics.net/) with *E*-value of 1 × 10^−5^ as the threshold [9,30]. Meanwhile, the tomato genome protein sequences were downloaded from SGN database and Hidden Markov Model (HMM) profiles of TCS characteristic domains, i.e., HisK (PF00512), HATPase (PF02518), HPt (PF01627), and REC (PF00072) were downloaded from Pfam (http://pfam.janelia.org/). Then we searched for TCS genes with HMMER 3.0 using the global HMM profile of these TCS characteristic domains with expected values less than 0.1. After removing redundant sequences, a total of 118 putative elements were identified. As a final quality check, each identified sequence using the two strategies above was subsequently confirmed using Pfam (http://pfam.janelia.org/) and SMART (http://smart.embl-heidelberg.de/) databases according to whether or not it possessed the structural characteristics and conserved domains of TCS elements, i.e., HisK, HATPase, REC, CHASE domain for cytokinin binding (CHASE), ethylene-binding domain (C2H4), and HPt domains. Tomato TCS homolog proteins in *Arabidopsis* were identified using BLASTP search against *Arabidopsis* databases of TAIR website (http://www.arabidopsis.org/) with default expected values. ExPASy [45] was used to calculate the molecular weights and isoelectric points (PIs) of putative tomato TCS proteins. Subcellular localizations were predicted using SubLoc v1.0 website [37].

### 4.2. Gene Structure Construction, Motif Analysis, and Phylogenetic Analysis

The exon–intron organizations of all tomato TCS genes were mapped using Gene Structure Display Server [46]. Each family motif was identified using the MEME program [47]. The predicted peptide sequences of the conserved domain in the TCS proteins were identified by employing the SMART database. Then multiple-sequence alignment for the predicted peptide sequences was generated using Clustal X v1.81 with default parameters [48]. The similarity of the tomato TCS proteins with those from *Arabidopsis*, rice, and tomato genome was calculated by DNAStar (Madison, WI, USA). Phylogenetic analysis was performed using MEGA 5.0 program by neighbor-joining (NJ) method with 1000 replicates of the bootstrap based on the full-length protein sequences [49].

### 4.3. Chromosomal Localization and Evolutionary Analysis of TCS Genes

All the TCS genes were assigned to the corresponding tomato chromosomes based on the SGN database. A pair of genes were identified as tandem duplicates if the genes both shared ≥40% amino acid sequence similarity and separated by fewer than five intervening genes [50]. PGDD [51] was adopted to perform synteny analysis and detect the segment duplications, as described in cucumber MADS gene family [50]. Full-length amino acid sequences were aligned using the ClustalW algorithm [52], and then *Ks* and *Ka* were calculated using the Codeml procedure of the PAML program [53]. Divergence time of the gene pairs was estimated using synonymous mutation rate of substitutions per synonymous site per year, as follows: *T* = *Ks*/2*x* (*x* = 6.56 × 10^−9^) [54].

### 4.4. Analysis of Putative Promoter Regions TCS Genes in Tomato

The upstream sequences (1.5 kb) of the initiation codon in TCS genomic DNA were obtained from Phytozome [44] as the putative promoter regions, and the *cis*-regulatory elements in the promoter regions were identified using PlantCARE website [55].

### 4.5. Subcellular Localization

The randomly selected TCS genes were amplified using gene-specific primers (Table S5) and cloned into the pFGC-EGFP plasmids by *Xba* I and *Bam*H I restriction sites under the control of the 35S cauliflower mosaic virus promoter (35S CaMV). The pFGC:GFP empty vector was used as control. The recombinant vectors were transformed into onion epidermal cells by particle bombardment using the Biolistic PDS-1000/He gene gun system (Bio-Rad, Hercules, CA, USA) [56]. After 16–18 h of incubation in darkness, the onion epidermal cell was plasmolyzed in 0.3 g·mL^−1^ sucrose for 5 min and the fluorescence of GFP was photographed by a Leica DMLE camera (Leica, Wetzlar, Germany).

### 4.6. Tomato Plant Growth and Treatments

Tomato (*S. lycopersicum* L.) cv. Micro-Tom from Tomato Genetics Resource Center (University of California, Davis, CA, USA) was used for expression analysis. The seedlings were grown in a growth chamber in temperature-controlled greenhouses of Zhejiang University under day/night temperatures of 28/20 ± 1 °C and light intensity of 250 μmol·m^−2^·s^−1^ with 16-h day length. Three-week-old tomato seedlings were used for abiotic stresses and exogenous hormone treatments. For cytokinin and ABA treatment, the seedlings were sprayed with 100 μM ZT and 100 μM ABA, respectively. The second true leaf on each plant was sampled at 0 (control), 1, 2, 4, and 8 h after spraying. To induce drought stress, the seedlings were transferred to filter paper and the leaves were collected at 0, 1, 2, 4, and 8 h. For high salt treatment, the nutrient solution was supplemented with 100 mM NaCl and the leaves were separately collected at 0, 1, 2, 4, and 8 h after treatment. All phytohormone and abiotic treatments were repeated three times and each treatment contained at least 20 seedlings. All materials were frozen at −75 °C until RNA isolation.

### 4.7. Expression Analysis of TCS Genes in Growth and Development Gene

The electronic expression data of tomato TCS genes in various organs were obtained by gene locus from the tomato eFP browser at http://bar.utoronto.ca [57]. The electronic expression profiles of all detected tomato TCS genes expressed in leaves, roots, unopened flower buds, fully opened flowers, and the fruits at six developmental stages (1 cm, 2 cm, and 3 cm fruit, mature green fruit, breaker fruit, and fruit at 10 days after breaker) were summarized and used to generate the heatmap with Multiple Array Viewer [58]. Tomato TCS genes were extracted for GO functional enrichment analysis (http://geneontology.org/) and KEGG pathway enrichment analysis [59] with default parameters.

### 4.8. RNA Isolation and qRT–PCR

The total RNA was extracted from the collected materials using TRIZOL reagent (Invitrogen, Karlsruhe, Germany) according to the manufacturer-recommended protocol. The first cDNA strand was generated from 1 μg of total RNA using the PrimerScript RT reagent kit (Takara, Otsu, Japan) according to the manufacturer’s instructions. Specific primers used in the qRT-PCR were designed by Primer 5 Software, and each primer was searched in the tomato database to ensure its specificity. The qRT-PCR reactions were performed on the CFX96 Real Time System machine (Bio-RAD, Hercules, CA, USA), programmed to heat for 30 s at 95 °C, followed by 40 cycles of 5 s at 95 °C and 45 s at 55 °C, and at the end, 1 cycle of 1 min at 95 °C, 30 s at 50 °C and 30 s at 95 °C. Two biological and three technical replicates for each sample were performed with 15 μL of reaction volume using the SYBR Premix Ex Taq kit (TOYOBO, Osaka, Japan). The tomato *SlUbi3* gene (GenBank accession number X58253) was selected as an internal control [60]. The relative gene expression level was calculated using the 2^−ΔΔ*C*t^ method. Heatmap was generated by Multiple Array Viewer using the relative expression data of each gene [58].

## 5. Conclusions

In our study, 20 HK(L)s, six HPs and 39 RRs were identified from tomato genome. Gene classification, gene structures, conserved domains, chromosome distribution, phylogenetic relationship, synteny relationship, gene duplication events, and subcellular localizations of the TCS genes were predicted and analyzed in detail. The tomato TCS elements showed significant sequence and domain conservation except type-B RRs. Gene duplication events mainly occurred in the RR family of tomato TCS genes. Both segment duplication and tandem duplication contributed to gene expansion. The subcellular localization of selected proteins displayed a diverse subcellular targeting and probably played divergent roles. Most TCS genes are predominantly expressed in tomato reproductive organs particularly in fruit development. Meanwhile, promoter analyses and qRT-PCR results indicated that almost all of TCSs could respond to various stresses and exogenous hormone treatments. The identification of tomato TCS elements would provide a more comprehensive sight and solid foundation to elucidate their roles in mediating hormone cross-talk and stress responses in further.

## Figures and Tables

**Table 1:** Summary of the two-component system (TCS) gene numbers identified in plants.

Species	HK(L)	HP (Pseudo-HP)	Type-A RR	Type-B RR	Type-C RR	Pseudo RR	Total	Reference
*Arabidopsis thaliana*	17 (9)	6 (1)	10	12	2	9	56	[4]
*Oryza sativa*	11 (3)	5 (3)	13	13	2	8	52	[7]
*Lotus japonicus*	14	7	7	11	1	5	40	[31]
*Glycine max*	36 (15)	13	18	15	3	13	98	[9]
*Zea mays*	11 (3)	9 (2)	16	9	3	11	59	[8]
*Physcomitrella patens*	18	3	7	5	2	4	39	[32]
*Triticum aestivum*	7	10	41	2	0	2	45	[30]
*Brassica rapa*	20 (9)	8 (1)	21	17	4	15	85	[10]
*Solanum lycopersicum*	20 (11)	6 (2)	7	23	1	8	65	–

HK(L), HP, and RR represent His-kinase or like, phosphotransfer, and response regulator protein, respectively.

**Table 2:** *Ks*, *Ka*, and *Ka/Ks* calculation and divergent time of the duplicated tomato TCS gene pairs.

Duplicated Gene Pairs	*Ks*	*Ka*	*Ka*/*Ks*	Duplicated Type	Purify Selection	Time (Mya *)
*SlHKL1/SlHKL4*	0.68	0.23	0.34	Segmental	Yes	52.31
*SlRR9/SlRR13*	0.79	0.38	0.48	Segmental	Yes	60.77
*SlRR22/SlRR23*	0.74	0.17	0.23	Segmental	Yes	56.92
*SlPRR1/SlPRR2*	0.60	0.21	0.35	Segmental	Yes	46.15
*SlRR16/SlRR18*	0.34	0.29	0.84	Tandem	Yes	26.15
*SlRR16/SlRR27*	0.32	0.23	0.71	Tandem	Yes	24.98
*SlRR16/SlRR28*	0.24	0.20	0.85	Tandem	Yes	18.48
*SlRR16/SlRR26*	0.20	0.19	0.99	Tandem	Yes	15.07
*SlRR18/SlRR27*	0.20	0.12	0.59	Tandem	Yes	15.08
*SlRR18/SlRR28*	0.31	0.26	0.84	Tandem	Yes	23.90
*SlRR18/SlRR26*	0.19	0.20	1.06	Tandem	No	14.80
*SlRR18/SlRR25*	0.21	0.18	0.84	Tandem	Yes	16.10
*SlRR25/SlRR27*	0.17	0.10	0.59	Tandem	Yes	13.45
*SlRR25/SlRR28*	0.08	0.06	0.79	Tandem	Yes	5.97
*SlRR25/SlRR26*	0.11	0.10	0.91	Tandem	Yes	8.28
*SlRR26/SlRR27*	0.35	0.33	0.96	Tandem	Yes	26.55
*SlRR26/SlRR28*	0.16	0.15	0.94	Tandem	Yes	12.34
*SlRR27/SlRR28*	0.11	0.06	0.58	Tandem	Yes	8.41

* Mya, million years ago.

## Supplementary Materials

Supplementary materials can be found at http://www.mdpi.com/1422-0067/17/8/1204/s1.

## References

[1] Mizuno T. (1997). Compilation of all genes encoding two-component phosphotransfer signal transducers in the genome of *Escherichia coli*. DNA Res..

[2] Stock A.M., Robinson V.L., Goudreau P.N. (2000). Two-component signal transduction. Annu. Rev. Biochem..

[3] Urao T., Yamaguchi-Shinozaki K., Shinozaki K. (2000). Two-component systems in plant signal transduction. Trends Plant Sci..

[4] Hwang I., Chen H.C., Sheen J. (2002). Two-component signal transduction pathways in *Arabidopsis*. Plant Physiol..

[5] Schaller G.E., Kieber J.J., Shiu S.H. (2008). Two-component signaling elements and histidyl-aspartyl phosphorelays. Arabidopsis Book.

[6] Grefen C., Harter K. (2004). Plant two-component systems: Principles, functions, complexity and cross talk. Planta.

[7] Pareek A., Singh A., Kumar M., Kushwaha H.R., Lynn A.M., Singla-Pareek S.L. (2006). Whole-genome analysis of *Oryza sativa* reveals similar architecture of two-component signaling machinery with *Arabidopsis*. Plant Physiol..

[8] Chu Z., Ma Q., Lin Y., Tang X., Zhou Y., Zhu S., Fan J., Cheng B. (2011). Genome-wide identification, classification, and analysis of two-component signal system genes in maize. Genet. Mol. Res..

[9] Mochida K., Yoshida T., Sakurai T., Yamaguchi-Shinozaki K., Shinozaki K., Tran L.S.P. (2010). Genome-wide analysis of two-component systems and prediction of stress-responsive two-component system members in soybean. DNA Res..

[10] Liu Z., Zhang M., Kong L., Lv Y., Zou M., Lu G., Cao J., Yu X. (2014). Genome-wide identification, phylogeny, duplication, and expression analyses of two-component system genes in Chinese cabbage (*Brassica rapa* ssp. *pekinensis*). DNA Res..

[11] Tran L.S.P., Urao T., Qin F., Maruyama K., Kakimoto T., Shinozaki K., Yamaguchi-Shinozaki K. (2007). Functional analysis of AHK1/ATHK1 and cytokinin receptor histidine kinases in response to abscisic acid, drought, and salt stress in Arabidopsis. Proc. Natl. Acad. Sci. USA.

[12] Wohlbach D.J., Quirino B.F., Sussman M.R. (2008). Analysis of the Arabidopsis histidine kinase ATHK1 reveals a connection between vegetative osmotic stress sensing and seed maturation. Plant Cell.

[13] Tran L.S.P., Shinozaki K., Yamaguchi-Shinozaki K. (2010). Role of cytokinin responsive two-component system in ABA and osmotic stress signalings. Plant Signal. Behav..

[14] Pham J., Liu J., Bennett M.H., Mansfield J.W., Desikan R. (2012). Arabidopsis histidine kinase 5 regulates salt sensitivity and resistance against bacterial and fungal infection. New Phytol..

[15] Kumar M.N., Jane W.N., Verslues P.E. (2013). Role of the putative osmosensor Arabidopsis histidine kinase1 in dehydration avoidance and low-water-potential response. Plant Physiol..

[16] Jeon J., Kim J. (2013). Arabidopsis response Regulator1 and Arabidopsis histidine phosphotransfer Protein2 (AHP2), AHP3, and AHP5 function in cold signaling. Plant Physiol..

[17] Nguyen K.H., Van Ha C., Nishiyama R., Watanabe Y., Leyva-González M.A., Fujita Y., Tran U.T., Li W., Tanaka M., Seki M. (2016). Arabidopsis type B cytokinin response regulators ARR1, ARR10, and ARR12 negatively regulate plant responses to drought. Proc. Natl. Acad. Sci. USA.

[18] Nishiyama R., Watanabe Y., Leyva-Gonzalez M.A., Van Ha C., Fujita Y., Tanaka M., Seki M., Yamaguchi-Shinozaki K., Shinozaki K. (2013). Arabidopsis AHP2, AHP3, and AHP5 histidine phosphotransfer proteins function as redundant negative regulators of drought stress response. Proc. Natl. Acad. Sci. USA.

[19] Nakamichi N., Kusano M., Fukushima A., Kita M., Ito S., Yamashino T., Saito K., Sakakibara H., Mizuno T. (2009). Transcript profiling of an Arabidopsis PSEUDO RESPONSE REGULATOR arrhythmic triple mutant reveals a role for the circadian clock in cold stress response. Plant Cell Physiol..

[20] Miyata S., Urao T., Yamaguchi-Shinozaki K., Shinozaki K. (1998). Characterization of genes for two-component phosphorelay mediators with a single HPt domain in Arabidopsis thaliana. FEBS Lett..

[21] Sun L., Zhang Q., Wu J., Zhang L., Jiao X., Zhang S., Zhang Z., Sun D., Lu T., Sun Y. (2014). Two rice authentic histidine phosphotransfer proteins, *OsAHP1* and *OsAHP2*, mediate cytokinin signaling and stress responses in rice. Plant Physiol..

[22] Feng W., Qin T., Yao W., Wen D., Zhang A., Tan M., Jiang M. (2015). *OsHK3* is a crucial regulator of abscisic acid signaling involved in antioxidant defense in rice. J. Integr. Plant Biol..

[23] Le D.T., Nishiyama R., Watanabe Y., Mochida K., Yamaguchi-Shinozaki K., Shinozaki K., Tran L.S. (2011). Genome-wide expression profiling of soybean two-component system genes in soybean root and shoot tissues under dehydration stress. DNA Res..

[24] Firon N., Pressman E., Meir S., Khoury R., Altahan L. (2012). Ethylene is involved in maintaining tomato (*Solanum lycopersicum*) pollen quality under heat-stress conditions. AOB Plants.

[25] D’Amico-Damiao V., Cruz F.J.R., Gavassi M.A., Santos D.M.M., Melo H.C., Carvalho R.F. (2015). Photomorphogenic modulation of water stress in tomato (*Solanum lycopersicum* L.): The role of phytochromes A, B1, and B2. J. Hortic. Sci. Biotechnol..

[26] Harry K., Denise T. (2002). The tomato ethylene receptor gene family: Form and function. Physiol. Plant..

[27] Pratt L.H., Cordonnier-Pratt M.M., Kelmenson P.M., Lazarova G.I., Kubota T., Alba R.M. (1997). The phytochrome gene family in tomato (*Solanum lycopersicum* L.). Plant Cell Environ..

[28] Tieman D.M., Taylor M.G., Ciardi J.A., Klee H.J. (2000). The Tomato Ethylene Receptors NR and LeETR4 Are Negative Regulators of Ethylene Response and Exhibit Functional Compensation within a Multigene Family. Proc. Natl. Acad. Sci. USA.

[29] Gupta S.K., Sharma S., Santisree P., Kilambi H.V., Appenroth K., Sreelakshmi Y., Sharma R. (2014). Complex and shifting interactions of phytochromes regulate fruit development in tomato. Plant Cell Environ..

[30] Gahlaut V., Mathur S., Dhariwal R., Khurana J.P., Tyagi A.K., Balyan H.S., Gupta P.K. (2014). A multi-step phosphorelay two-component system impacts on tolerance against dehydration stress in common wheat. Funct. Integr. Genom..

[31] Ishida K., Niwa Y., Yamashino T., Mizuno T. (2009). A genome-wide compilation of the two-component systems in *Lotus japonicus*. DNA Res..

[32] Ishida K., Yamashino T., Nakanishi H., Mizuno T. (2010). Classification of the genes involved in the two-component system of the moss *Physcomitrella patens*. Biosci. Biotechnol. Biochem..

[33] Rockwell N.C., Su Y.S., Lagarias J.C. (2006). Phytochrome structure and signaling mechanisms. Annu. Rev. Plant Biol..

[34] Hayama R., Coupland G. (2003). Shedding light on the circadian clock and the photoperiodic control of flowering. Curr. Opin. Plant Biol..

[35] Pan Y., Bradley G., Pyke K., Ball G., Lu C., Fray R., Marshall A., Jayasuta S., Baxter C., van Wijk R. (2013). Network inference analysis identifies an APRR2-like gene linked to pigment accumulation in tomato and pepper fruits. Plant Physiol..

[36] Pils B., Heyl A. (2009). Unraveling the Evolution of Cytokinin Signaling. Plant Physiol..

[37] SubLoc. http://www.bioinfo.tsinghua.edu.cn/SubLoc/eu_predict.htm.

[38] Dortay H., Gruhn N., Pfeifer A., Schwerdtner M., Schmülling T., Heyl A. (2008). Toward an interaction map of the two-component signaling pathway of Arabidopsis thaliana. J. Proteom. Res..

[39] Consortium T.T.G. (2012). The tomato genome sequence provides insights into fleshy fruit evolution. Nature.

[40] Blanc G., Wolfe K.H. (2004). Widespread paleopolyploidy in model plant species inferred from age distributions of duplicate genes. Plant Cell.

[41] Hanada K., Zou C., Lehti-Shiu M.D., Shinozaki K., Shiu S.H. (2008). Importance of lineage-specific expansion of plant tandem duplicates in the adaptive response to environmental stimuli. Plant Physiol..

[42] Ciardi J.A., Tieman D.M., Jones J.B., Klee H.J. (2001). Reduced expression of the tomato ethylene receptor gene *LeETR4* enhances the hypersensitive response to *Xanthomonas* campestris pv. vesicatoria. Mol. Plant Microb. Interact..

[43] Poór P., Kovács J., Borbély P., Takács Z., Szepesi Á., Tari I. (2015). Salt stress-induced production of reactive oxygen-and nitrogen species and cell death in the ethylene receptor mutant *Never ripe* and wild type tomato roots. Plant Physiol. Biochem..

[44] Phytozome. http://phytozome.jgi.doe.gov/pz/portal.html.

[45] ExPASy. http://web.expasy.org/compute_pi/.

[46] Gene Structure Display Server. http://gsds.cbi.pku.edu.cn.

[47] MEME. http://meme-suite.org/tools/meme.

[48] Thompson J.D., Gibson T.J., Plewniak F., Jeanmougin F., Higgins D.G. (1997). The CLUSTAL_X windows interface: Flexible strategies for multiple sequence alignment aided by quality analysis tools. Nucleic Acids Res..

[49] Tamura K., Peterson D., Peterson N., Stecher G., Nei M., Kumar S. (2011). MEGA5: Molecular evolutionary genetics analysis using maximum likelihood, evolutionary distance, and maximum parsimony methods. Mol. Biol. Evol..

[50] Hu L., Liu S. (2012). Genome-wide analysis of the MADS-box gene family in cucumber. Genome.

[51] PGDD. http://chibba.agtec.uga.edu/duplication/.

[52] ClustalW. http://www.genome.jp/tools/clustalw/.

[53] PAML. http://www.bork.embl.de/pal2nal/.

[54] Yuan S., Xu B., Jing Z., Zheni X., Qiang C., Zhimin Y., Qingsheng C., Bingru H. (2015). Comprehensive analysis of CCCH-type zinc finger family genes facilitates functional gene discovery and reflects recent allopolyploidization event in tetraploid switchgrass. BMC Genom..

[55] PlantCARE. http://bioinformatics.psb.ugent.be/webtools/plantcare/html/search_CARE.html.

[56] Gan C. (1989). Gene gun accelerates DNA-coated particles to transform intact-cells. Scientist.

[57] Tomato eFP Browser. http://bar.utoronto.ca/efp_tomato/cgi-bin/efpWeb.cgi.

[58] Saeed A.I., Bhagabati N.K., Braisted J.C., Wei L., Sharov V., Howe E.A., Li J., Thiagarajan M., White J.A., Quackenbush J. (2006). TM4 Microarray Software Suite. Method Enzymol..

[59] KEGG. http://www.genome.jp/kaas-bin/kaas_main.

[60] Wu J., Liu S., He Y., Guan X., Zhu X., Cheng L., Wang J., Lu G. (2012). Genome-wide analysis of SAUR gene family in Solanaceae species. Gene.

