# Supplementary material for: Genome-Wide Identification and Expression Analysis of Two-Component System Genes in Tomato
Source: Int J Mol Sci. 2016 Jul 26;17(8):1204. doi: 10.3390/ijms17081204 (PMC5000602; doi:10.3390/ijms17081204)
Supplement: Supplementary file 1 [file ijms-17-01204-s001.zip › ijms-136003-Supplementary Materials/ijms-136003-supplementary.pdf]

Supplementary Materials: Genome-Wide Identification and Expression Analysis of Two-Component System Genes in Tomato

Yanjun He, Xue Liu, Lei Ye, Changtian Pan, Lifei Chen, Tao Zou and Gang Lu

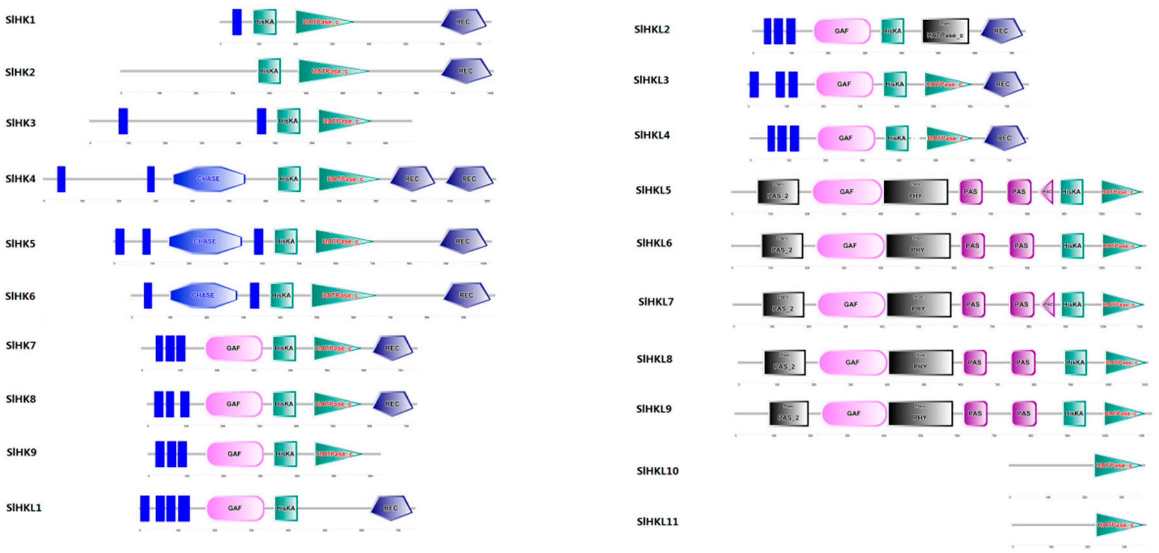

Figure S1. Domain structures of SlHK(L)s in tomato. Domain structures were analyzed by SMART online tool and drawn according to their original location and size. TM, transmembrane region; HisKA, Histidine kinase domain; HATPase, Histidine kinase-like HATPases; Rec, receiver domain; CHASE, cyclase/histidine kinase-associated sensory extracellular domain; GAF, cyclic GMP adenylyl cyclase FhlA domain; PHY, chromophore-binding domain; PAS, Per/Arndt/Sim folds.

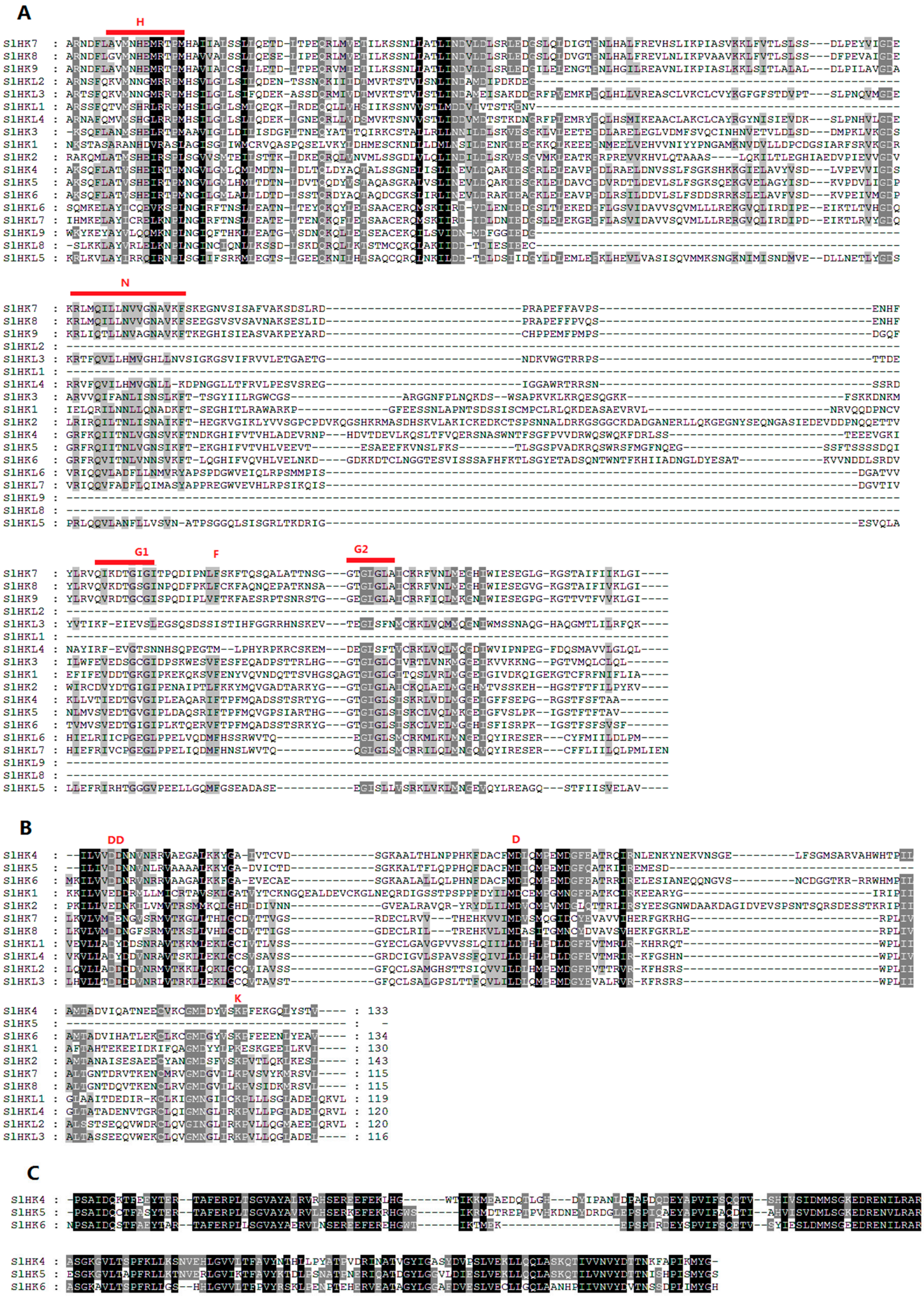

Figure S2. Amino acid sequence alignment of SlHK(L)s in tomato. Histidine kinase (like) (**A**); receiver (**B**); and cyclase/histidine kinase-associated sensory extracellular (CHASE) (**C**) domains from SlHK(L) proteins in tomato were aligned by the Clustal X program.

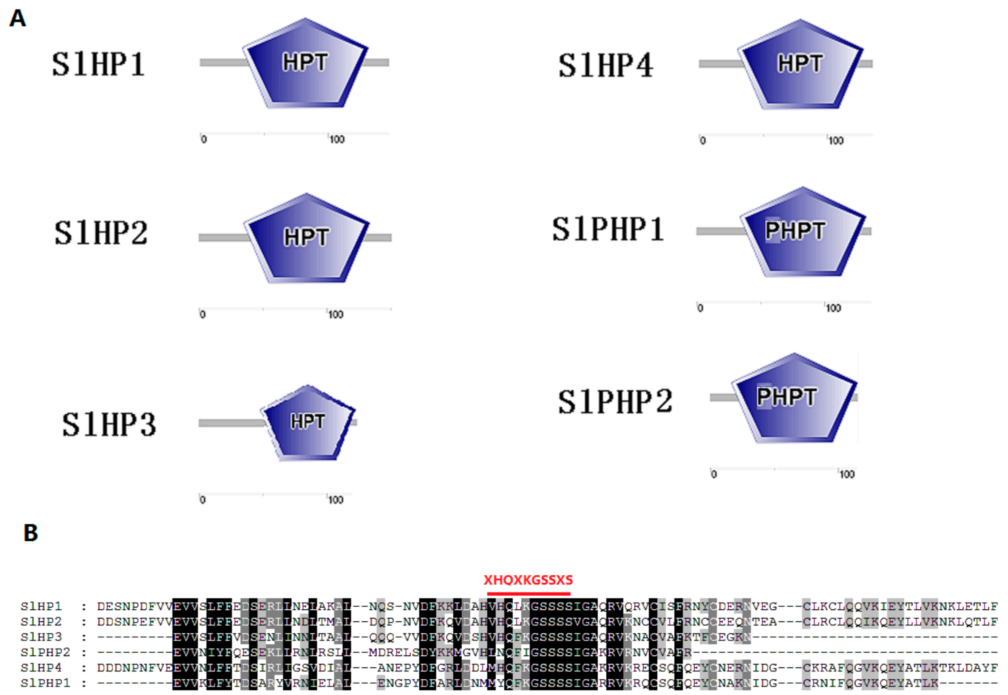

Figure S3. Domain structures and alignment of deduced amino acid sequences of SlHP proteins in tomato. (**A**) Domain structures were analyzed by SMART online tool and drawn according to their original location and size. HPt, His-containing phosphotransfer domain; PHPt, pseudo His-containing phosphotransfer domain; (**B**) Sequences were aligned by the Clustal X program. The Hpt domain has been highlighted by red line. The conserved XHQXKGSSXS motif was also marked above the alignment.

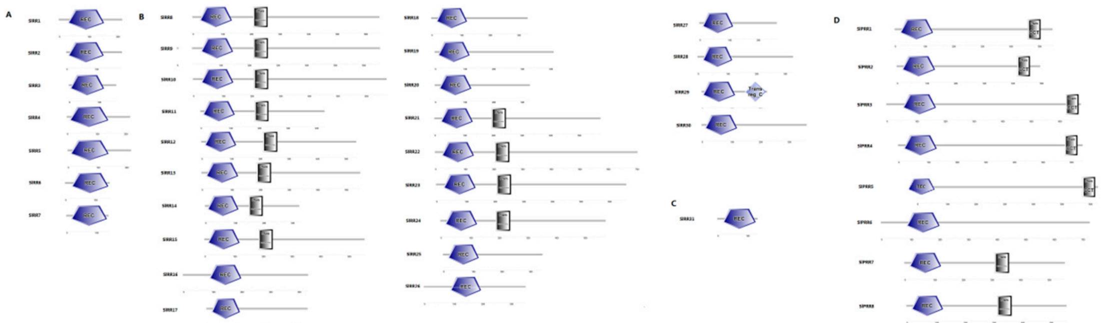

Figure S4. Domain structures of SlRR genes in tomato. Domain structures were analyzed by SMART online tool and drawn according to their original location and size. Rec, receiver domain; PRec, pseudo receiver domain; Myb, Myb DNA-binding domain; CCT, plant-specific CCT motif. (**A**–**D**) represent the domain structures of type-A RR, type-B RR, type-C RR, and PRR, respectively.

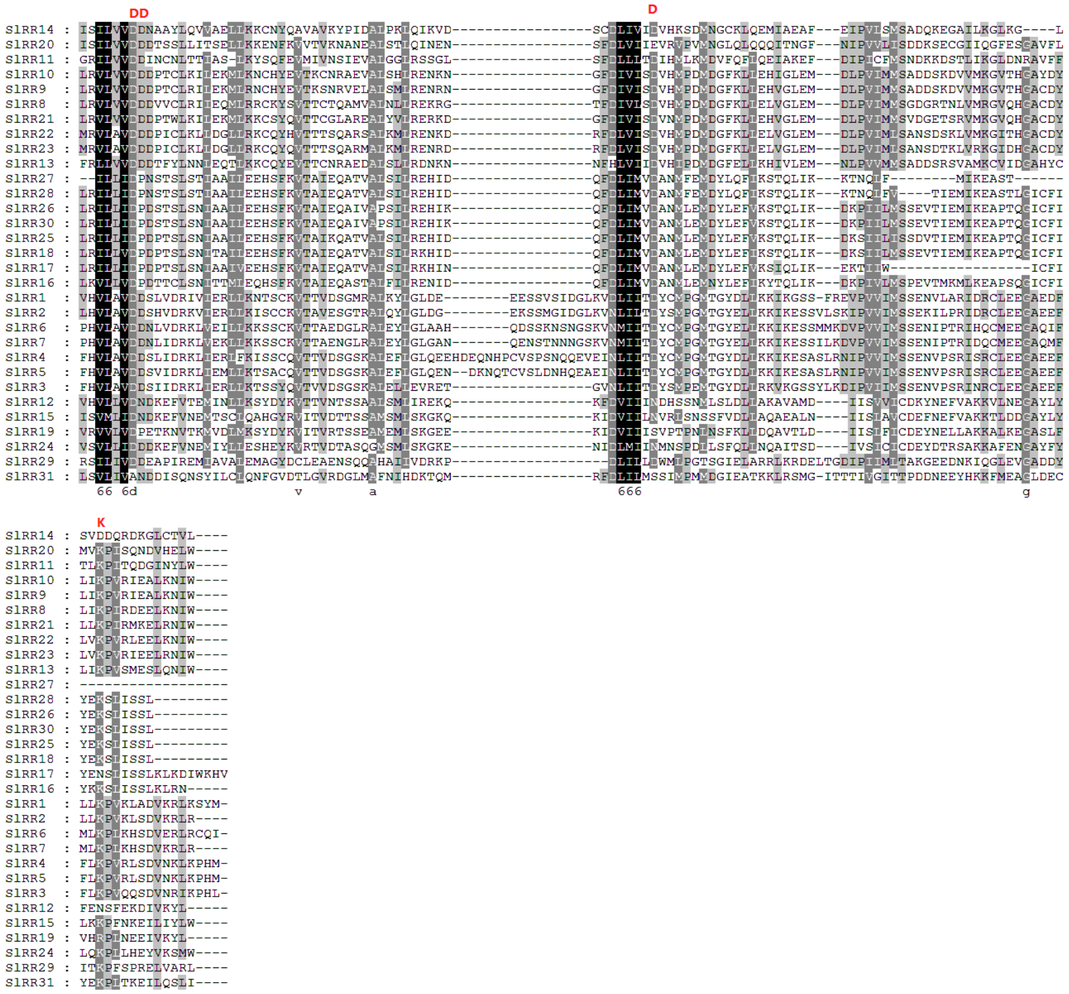

Figure S5. Alignment of deduced amino acids sequences of REC domain from RR proteins in tomato. Sequences were aligned by the Clustal X program and the highly conserved amino acids are highlighted.

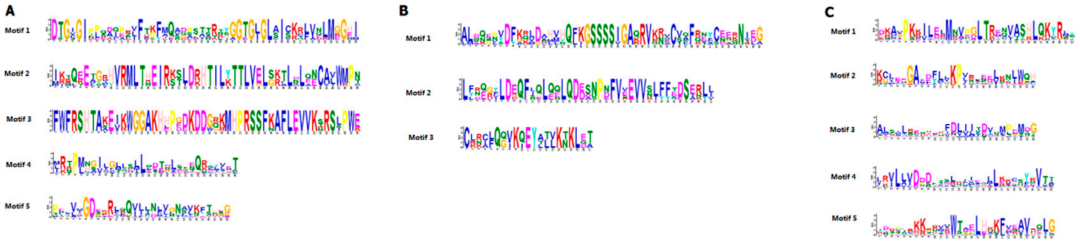

Figure S6. The conserved motif LOGO of HK(L) (**A**); HP (**B**); and RR (**C**) genes in tomato.

Table S4. The KEGG pathway enrichment analysis for tomato TCS genes.

Pathway ID	Pathway	KO List	Gene lIst
ko02020	Two-component system	ko:K07657	*SlRR29*
ko04075	Plant hormone signal transduction	ko:K14489	*SlHK4, SlHK5, SlHK6*
		ko:K14490	*SlHP1, SlHP2, SlHP3, SlHP4, SlPHP1, SlPHP2*
		ko:K14491	*SlRR8, SlRR9, SlRR10*
		ko:K14492	*SlRR1, SlRR2, SlRR3, SlRR4, SlRR5, SlRR6, SlRR7*
		ko:K14509	*SlHK7, SlHK8, SlHK9, SlHKL1, SlHKL2, SlHKL3, SlHKL4*
ko04712	Circadian rhythm–plant	ko:K12120	*SlHKL5, SlHKL8*
		ko:K12121	*SlHKL6, SlHKL7*
		ko:K12127	*SlPRR1, SlPRR2*
		ko:K12129	*SlPRR5, SlPRR6*
		ko:K12130	*SlPRR3, SlPRR4*

Table S5. The expression patterns of tomato TCS genes in response to various hormone (ZT (**A**) and ABA (**B**)) and stresses (drought (**C**) and salt (**D**)). The second true leaves were collected at 0, 1, 2, 4, and 8 h after the onset of treatments. Asterisks on the top of bars (SE values) indicate statistically significant difference between the compared pairs (* $p < 0.05$; ** $p < 0.01$).

(**A**)

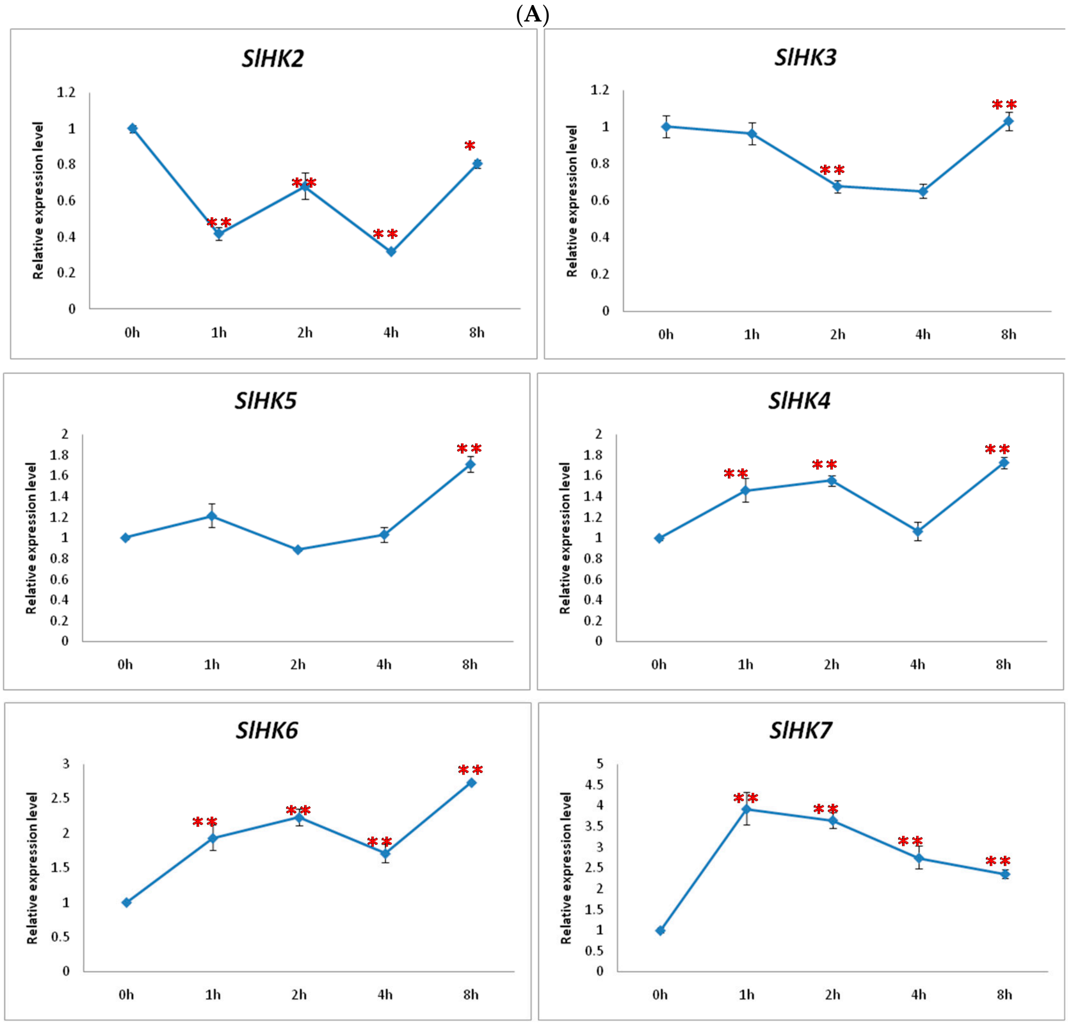

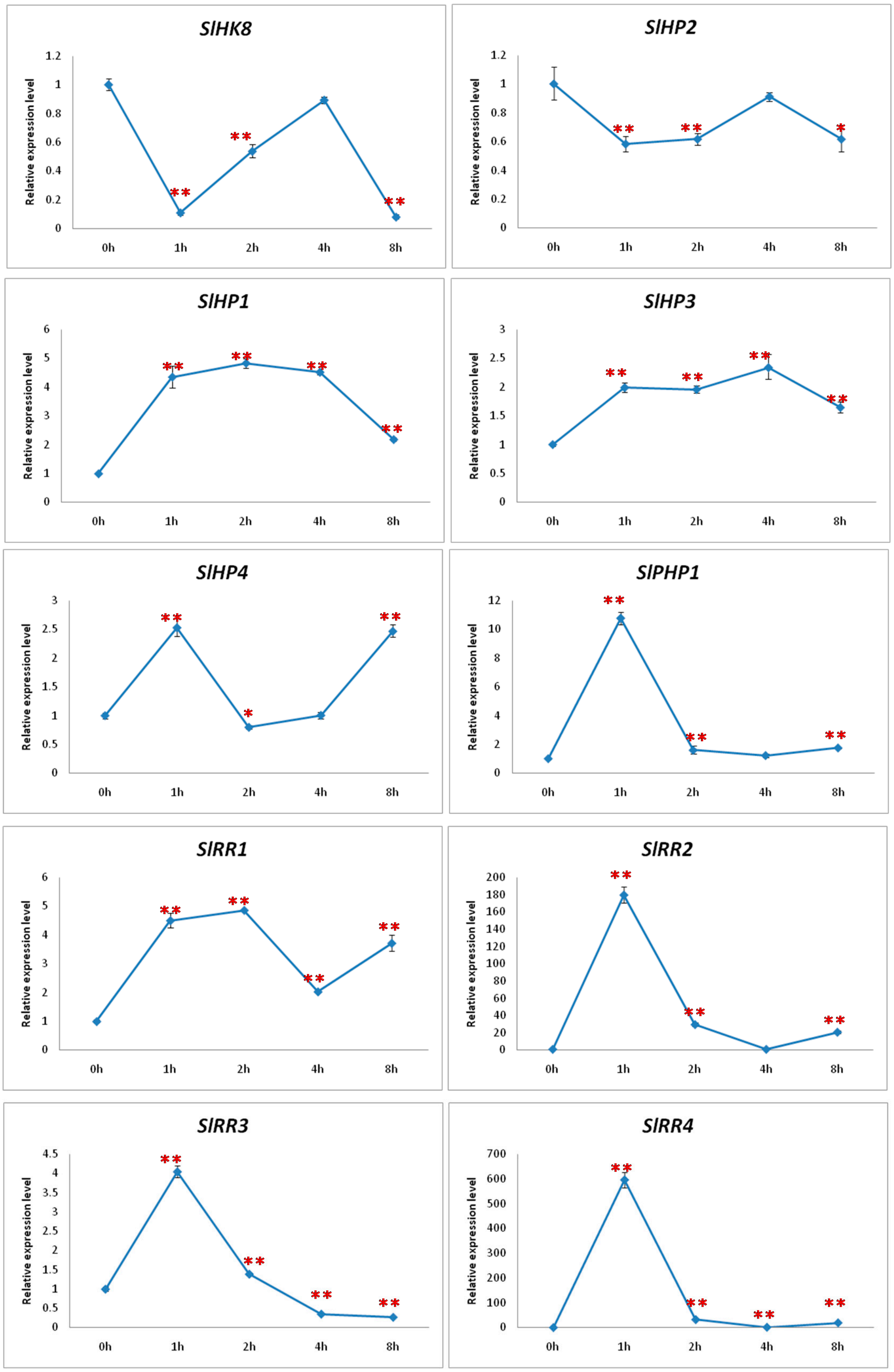
SlHK8
SlHP2
SlHP1
SlHP3
SlHP4
SlPHP1
SlRR1
SlRR2
SlRR3
SlRR4
Relative expression level
0h
1h
2h
4h
8h

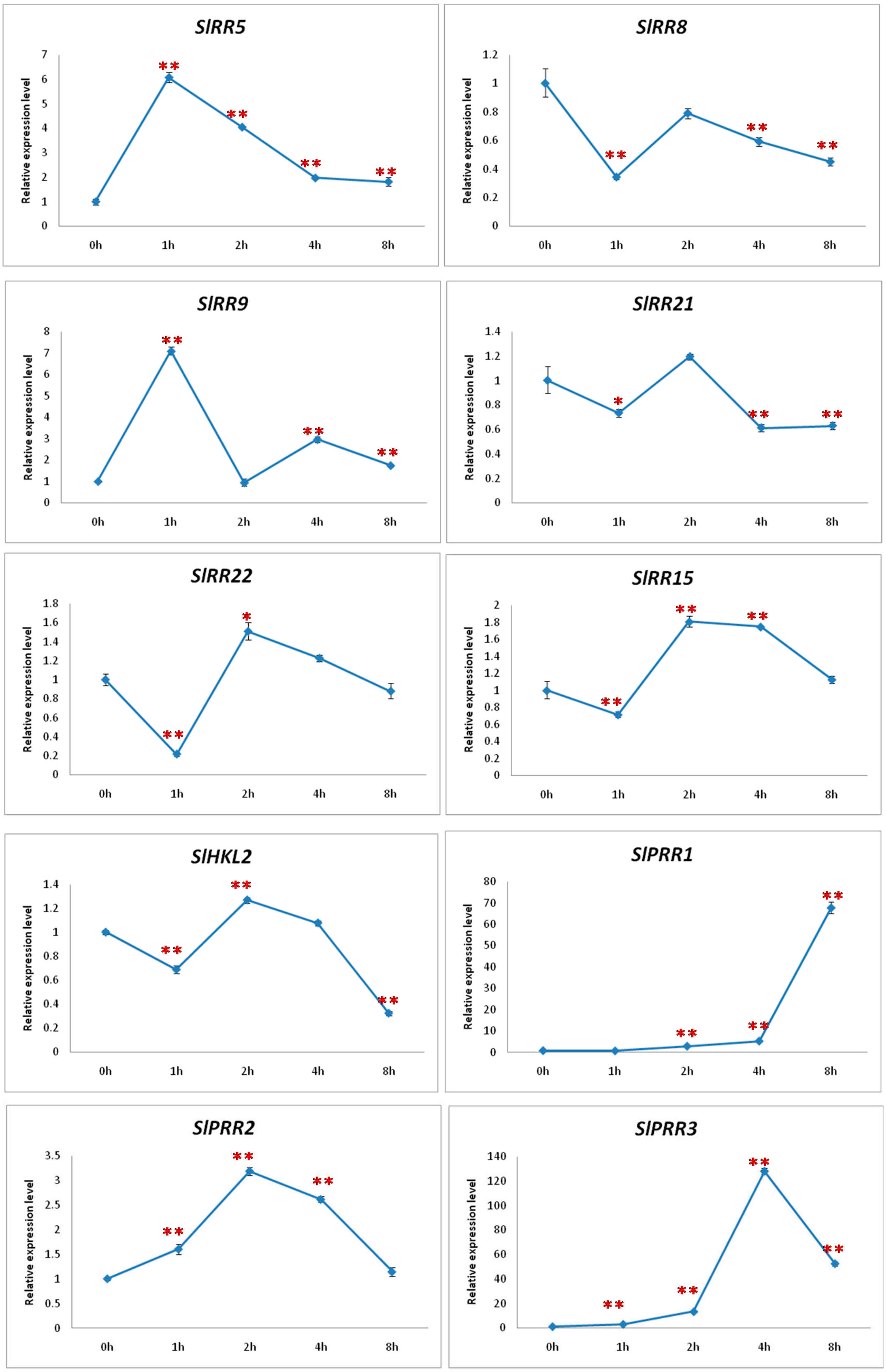
SlRR5
SlRR8
SlRR9
SlRR21
SlRR22
SlRR15
SlHKL2
SlPRR1
SlPRR2
SlPRR3
Relative expression level
0h
1h
2h
4h
8h

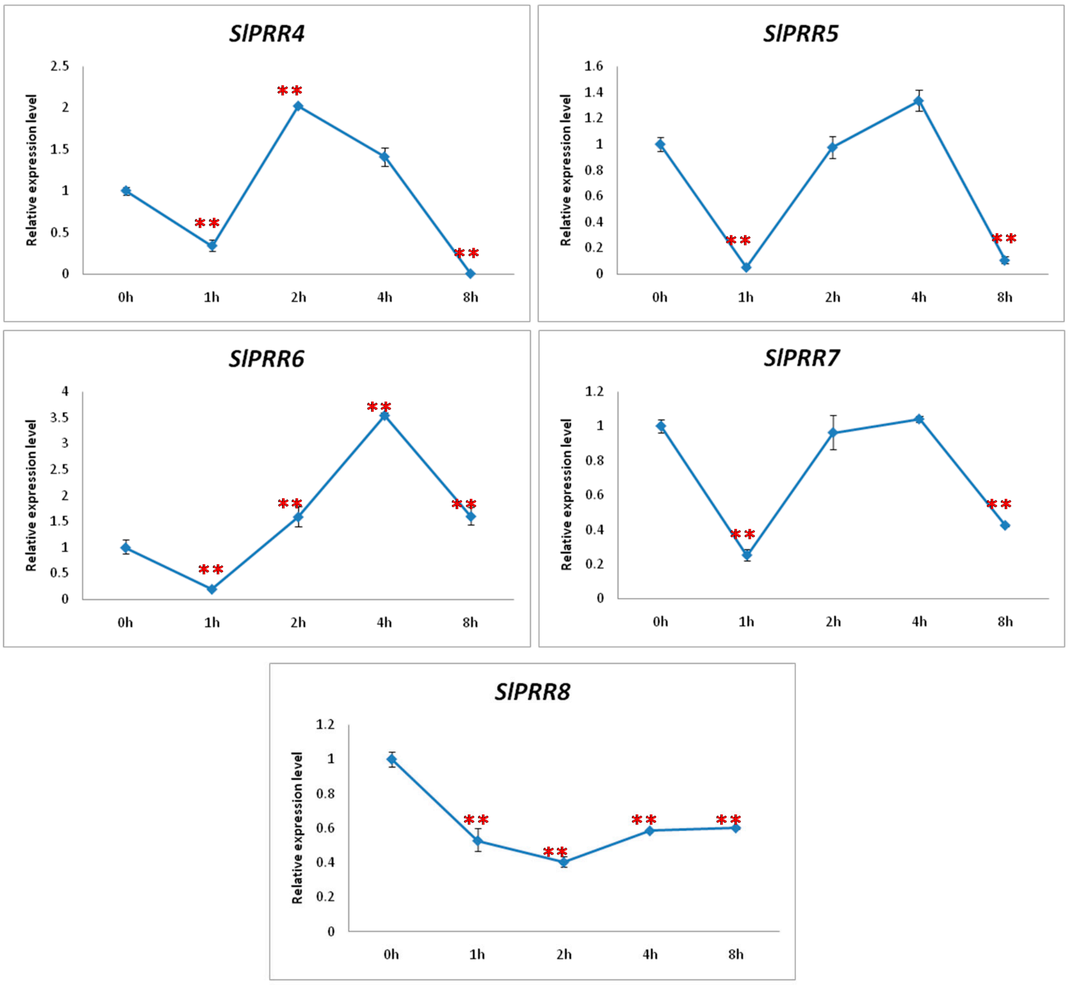
SlPRR4
SlPRR5
SlPRR6
SlPRR7
SlPRR8
Relative expression level
0h
1h
2h
4h
8h

(**B**)

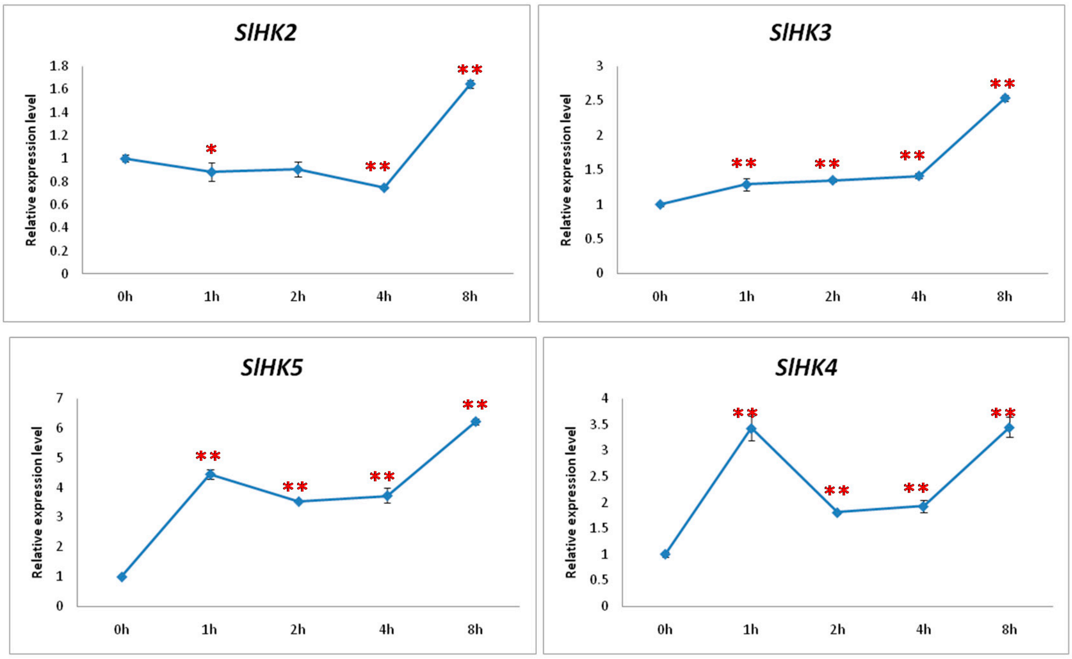
SlHK2
SlHK3
SlHK5
SlHK4
Relative expression level
0h
1h
2h
4h
8h

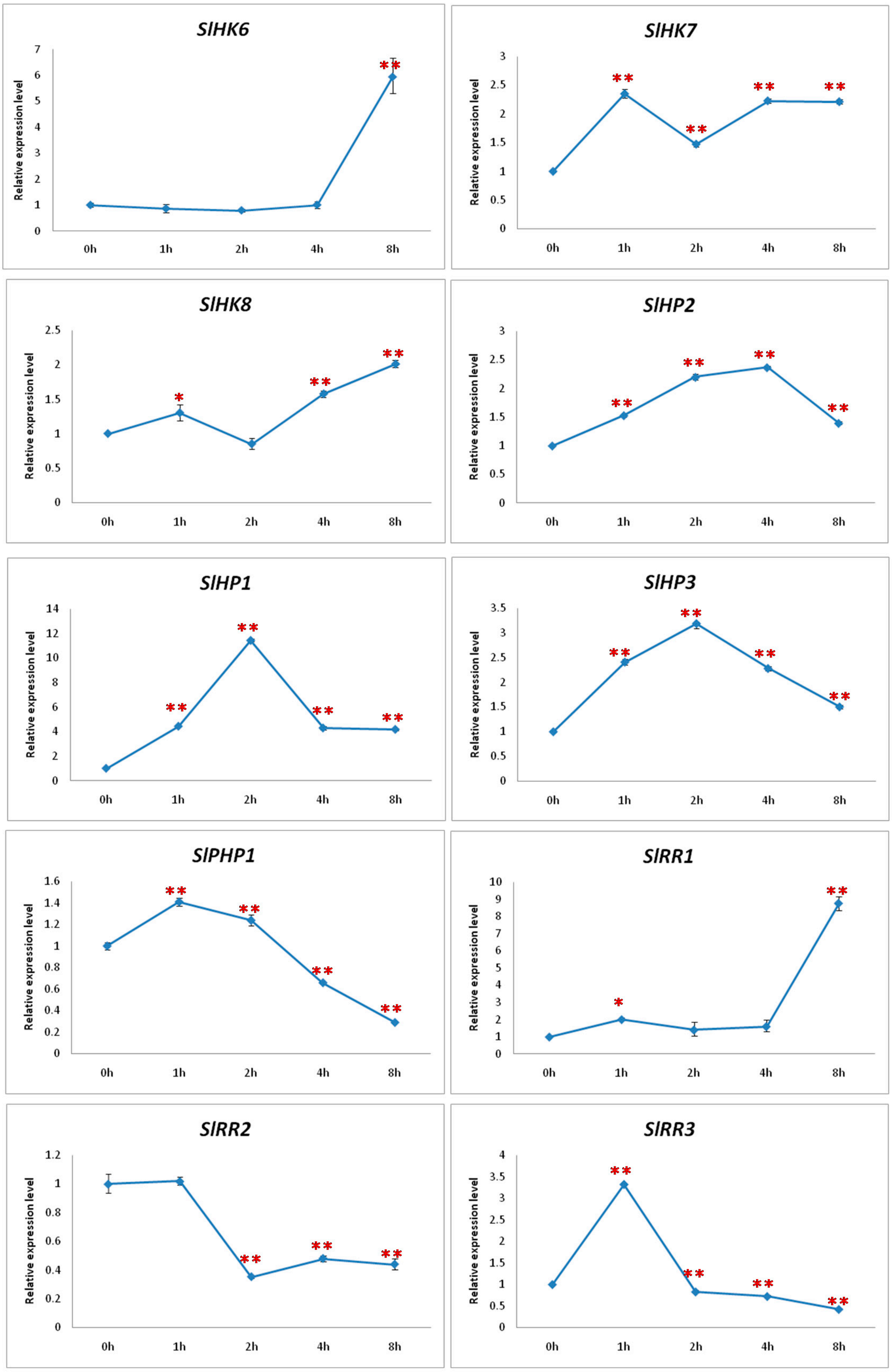
SlHK6
SlHK7
SlHK8
SlHP2
SlHP1
SlHP3
SlPHP1
SlRR1
SlRR2
SlRR3
Relative expression level
0h
1h
2h
4h
8h

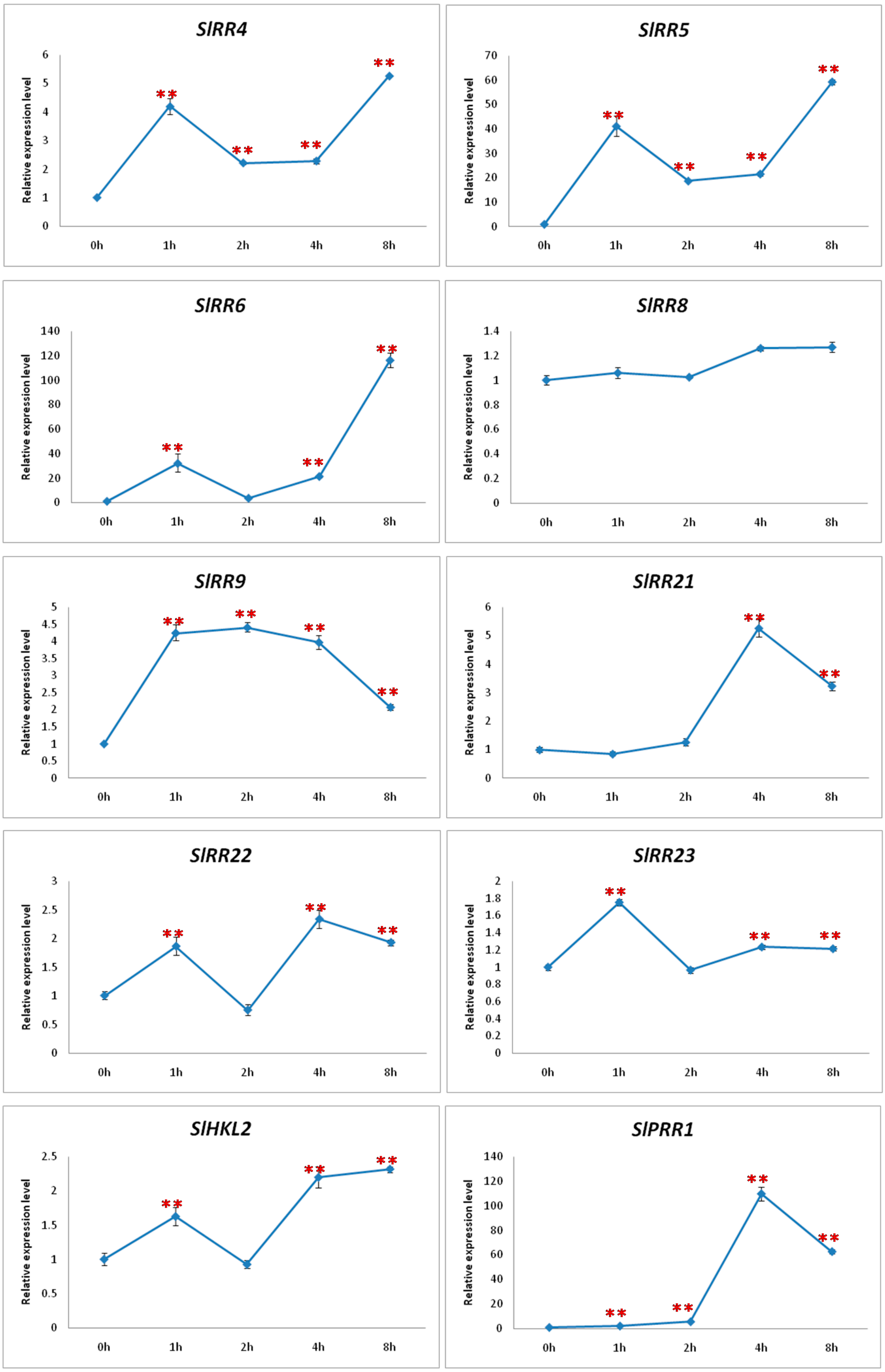
SlRR4
SlRR5
SlRR6
SlRR8
SlRR9
SlRR21
SlRR22
SlRR23
SlHKL2
SlPRR1
Relative expression level
0h
1h
2h
4h
8h
**

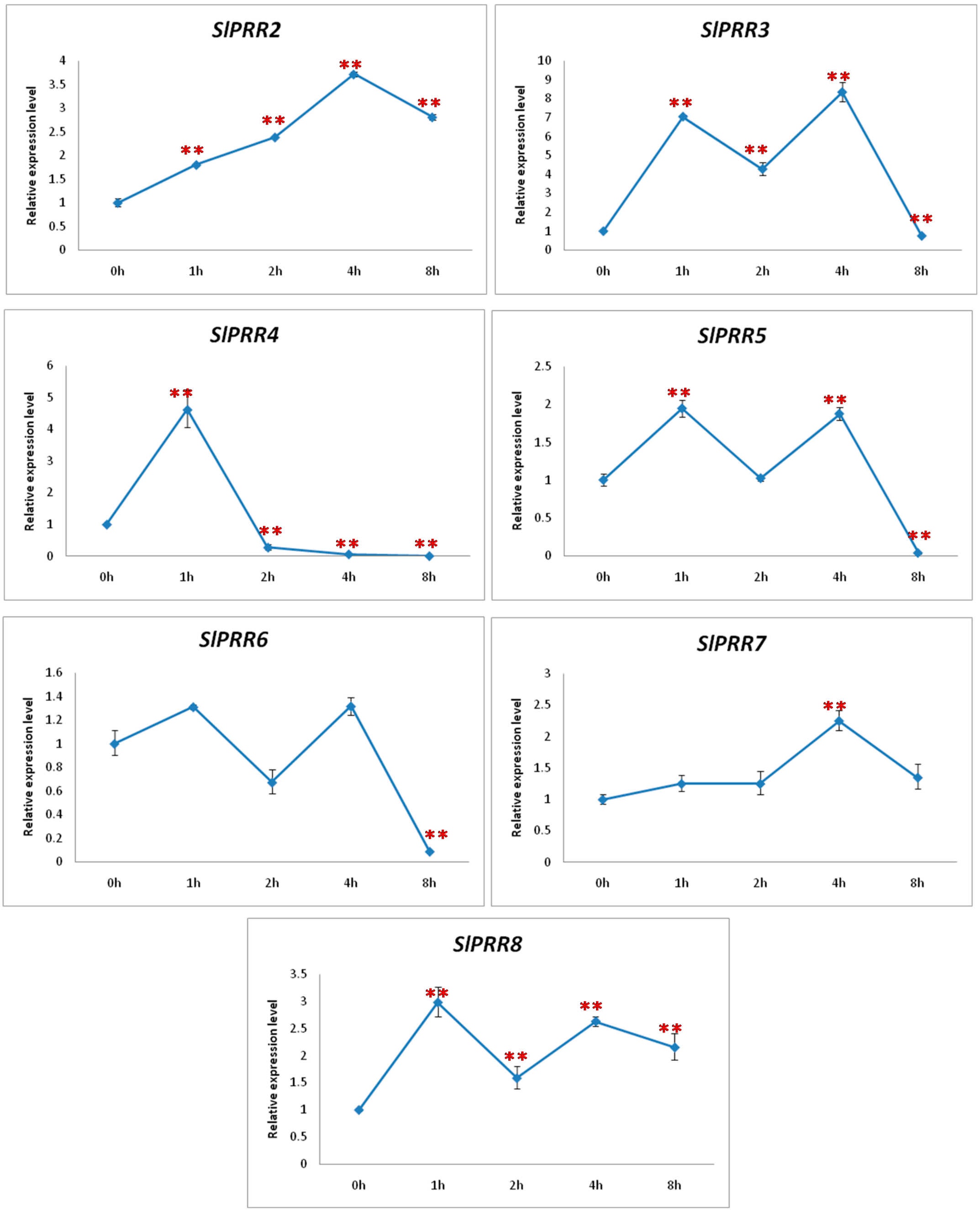

SlPRR2
Relative expression level
0 0.5 1 1.5 2 2.5 3 3.5 4
0h 1h 2h 4h 8h
SlPRR3
Relative expression level
0 1 2 3 4 5 6 7 8 9 10
0h 1h 2h 4h 8h
SlPRR4
Relative expression level
0 1 2 3 4 5 6
0h 1h 2h 4h 8h
SlPRR5
Relative expression level
0 0.5 1 1.5 2 2.5
0h 1h 2h 4h 8h
SlPRR6
Relative expression level
0 0.2 0.4 0.6 0.8 1 1.2 1.4 1.6
0h 1h 2h 4h 8h
SlPRR7
Relative expression level
0 0.5 1 1.5 2 2.5 3
0h 1h 2h 4h 8h
SlPRR8
Relative expression level
0 0.5 1 1.5 2 2.5 3 3.5
0h 1h 2h 4h 8h

(**C**)

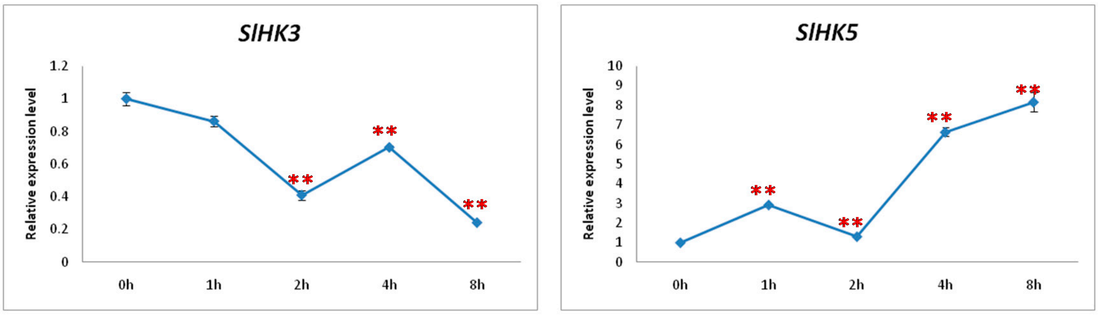

SlHK3
Relative expression level
0 0.2 0.4 0.6 0.8 1 1.2
0h 1h 2h 4h 8h
SlHK5
Relative expression level
0 1 2 3 4 5 6 7 8 9 10
0h 1h 2h 4h 8h

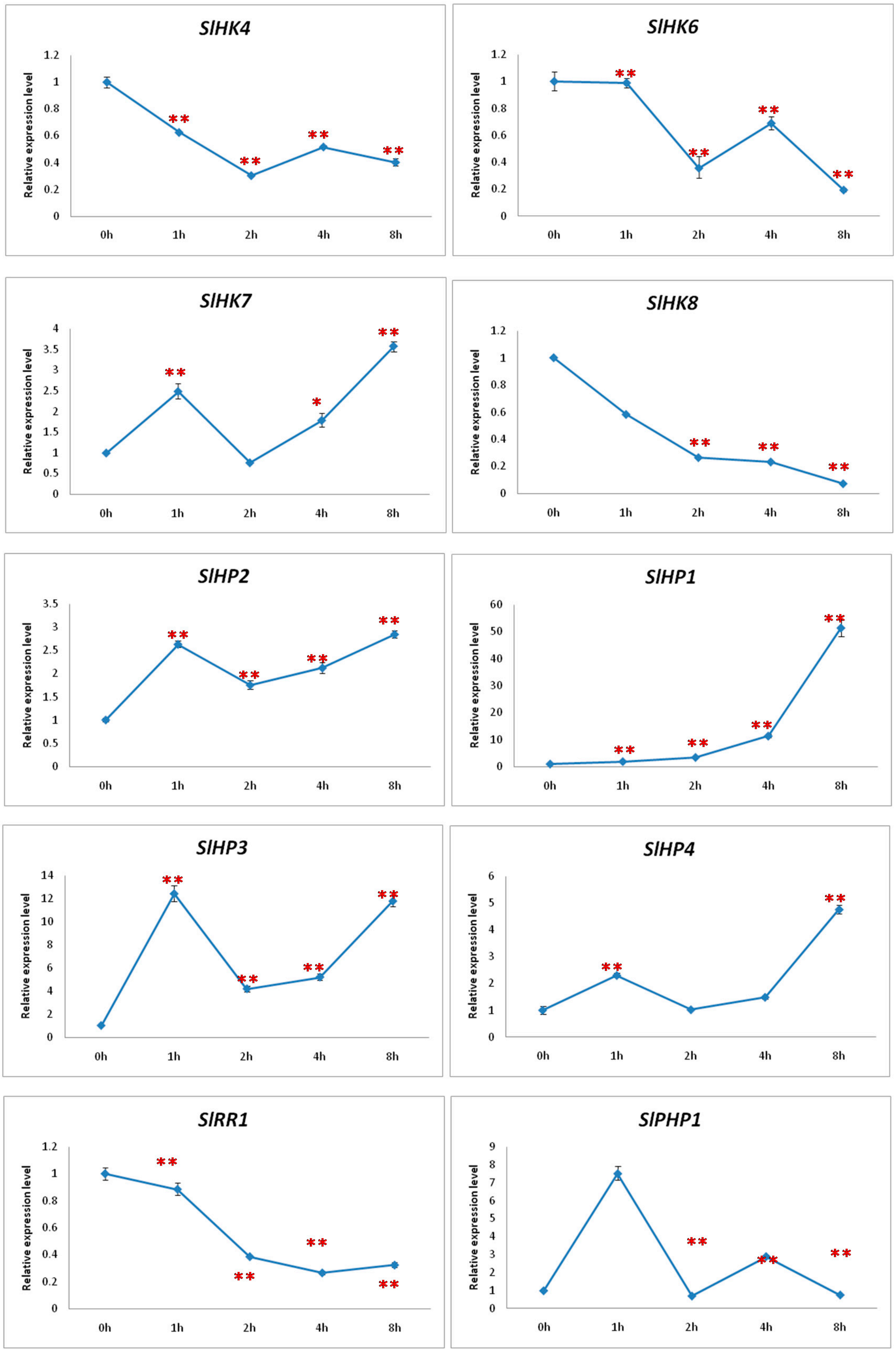
SlHK4
SlHK6
SlHK7
SlHK8
SlHP2
SlHP1
SlHP3
SlHP4
SlRR1
SlPHP1
Relative expression level
0h
1h
2h
4h
8h

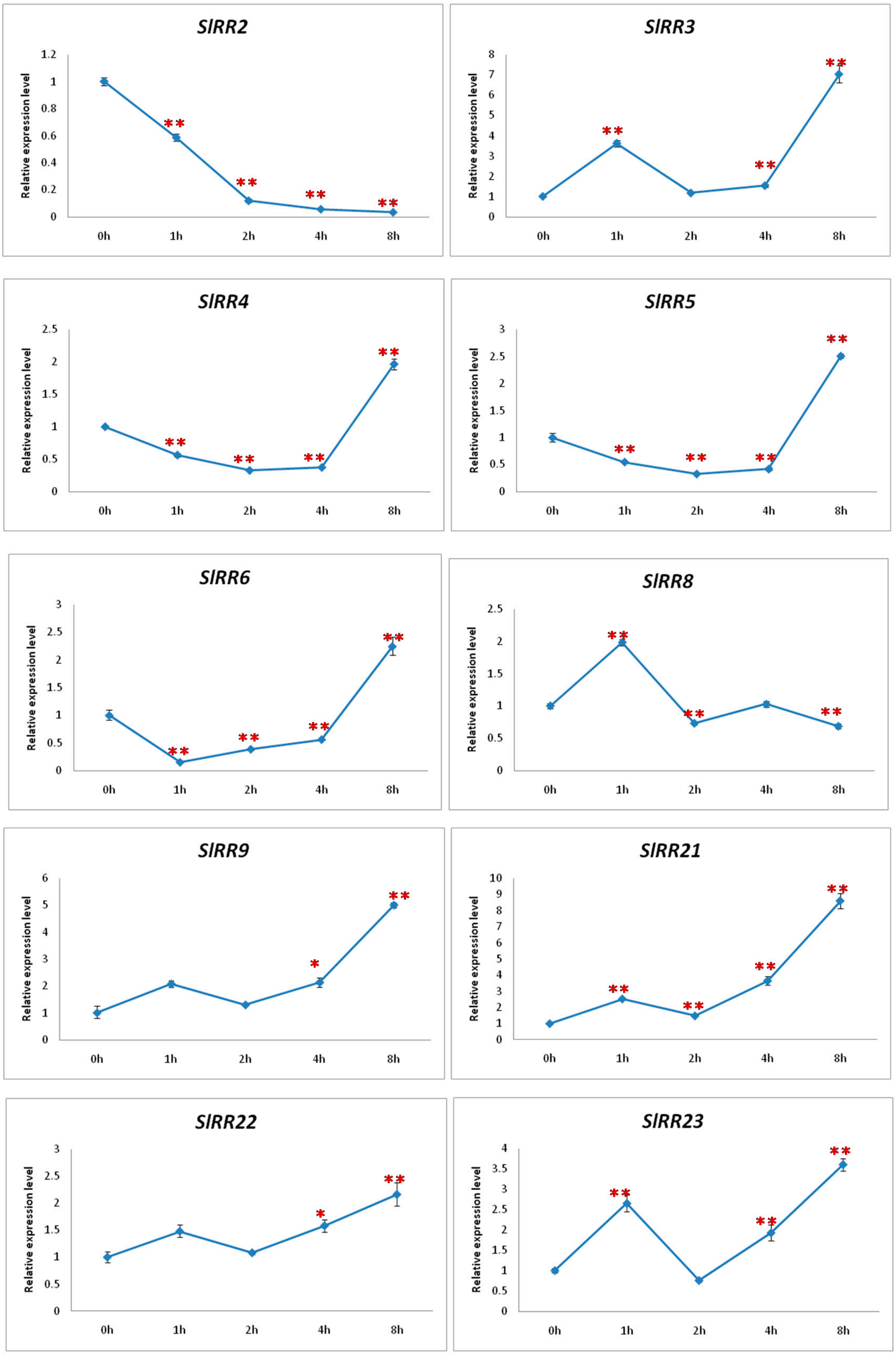

SlRR2
SlRR3
SlRR4
SlRR5
SlRR6
SlRR8
SlRR9
SlRR21
SlRR22
SlRR23
Relative expression level
0h
1h
2h
4h
8h

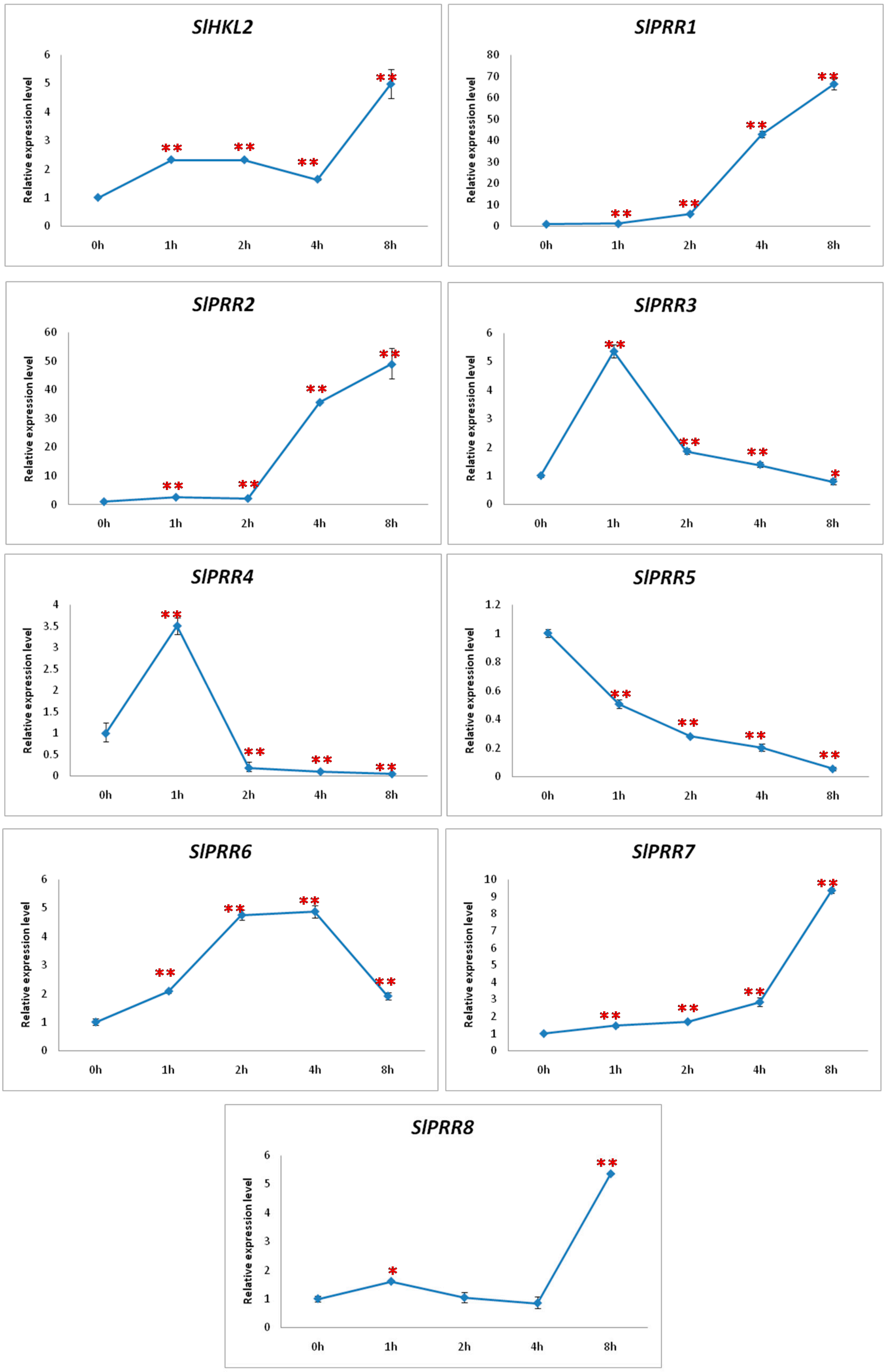

SlHKL2
Relative expression level
0 1 2 3 4 5 6
0h 1h 2h 4h 8h
SlPRR1
Relative expression level
0 10 20 30 40 50 60 70 80
0h 1h 2h 4h 8h
SlPRR2
Relative expression level
0 10 20 30 40 50 60
0h 1h 2h 4h 8h
SlPRR3
Relative expression level
0 1 2 3 4 5 6
0h 1h 2h 4h 8h
SlPRR4
Relative expression level
0 0.5 1 1.5 2 2.5 3 3.5 4
0h 1h 2h 4h 8h
SlPRR5
Relative expression level
0 0.2 0.4 0.6 0.8 1 1.2
0h 1h 2h 4h 8h
SlPRR6
Relative expression level
0 1 2 3 4 5 6
0h 1h 2h 4h 8h
SlPRR7
Relative expression level
0 1 2 3 4 5 6 7 8 9 10
0h 1h 2h 4h 8h
SlPRR8
Relative expression level
0 1 2 3 4 5 6
0h 1h 2h 4h 8h

(**D**)

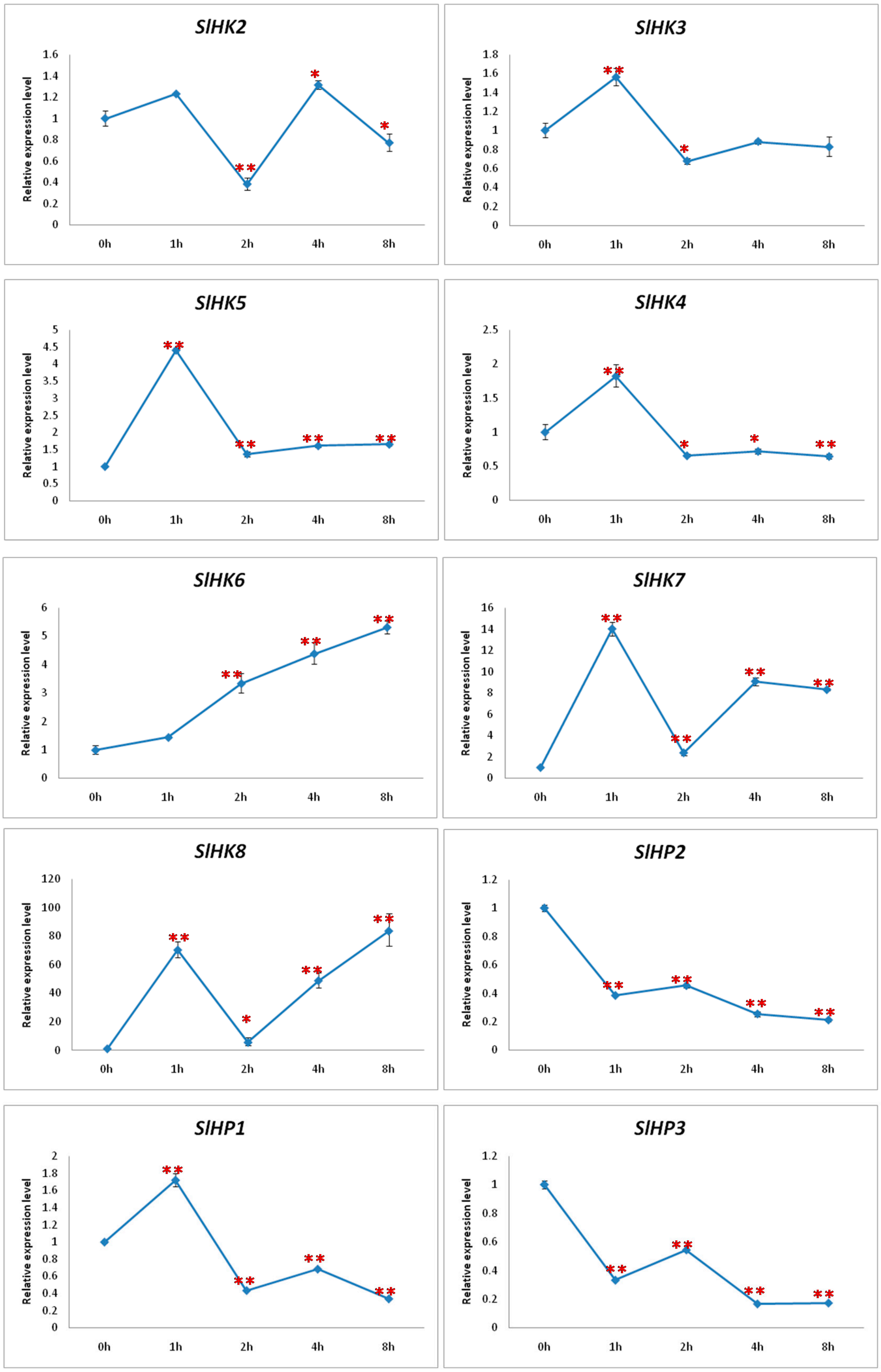
SlHK2
SlHK3
SlHK5
SlHK4
SlHK6
SlHK7
SlHK8
SlHP2
SlHP1
SlHP3
Relative expression level
0h
1h
2h
4h
8h

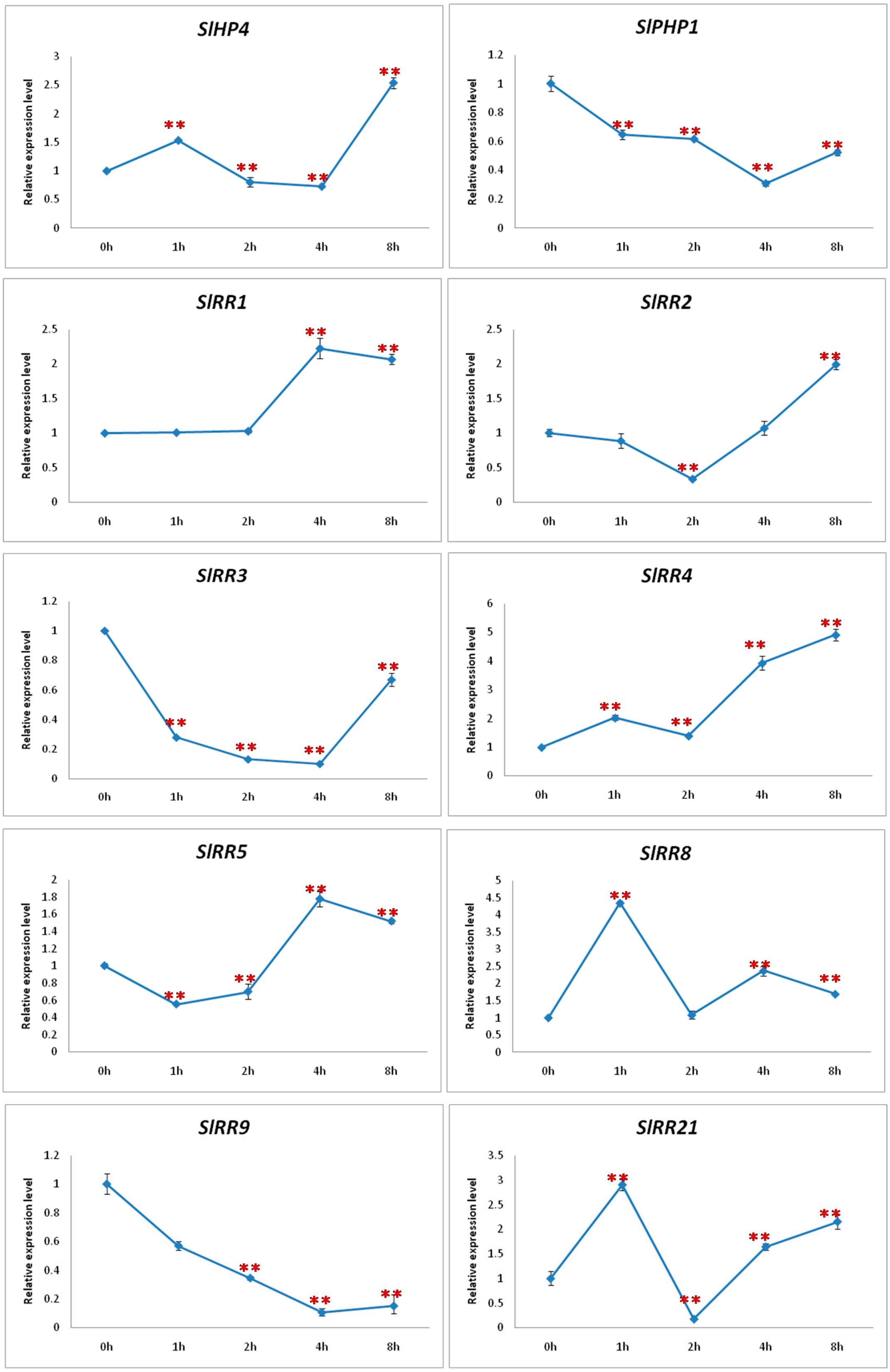
SlHP4
SlPHP1
SlRR1
SlRR2
SlRR3
SlRR4
SlRR5
SlRR8
SlRR9
SlRR21
Relative expression level
0h
1h
2h
4h
8h
**

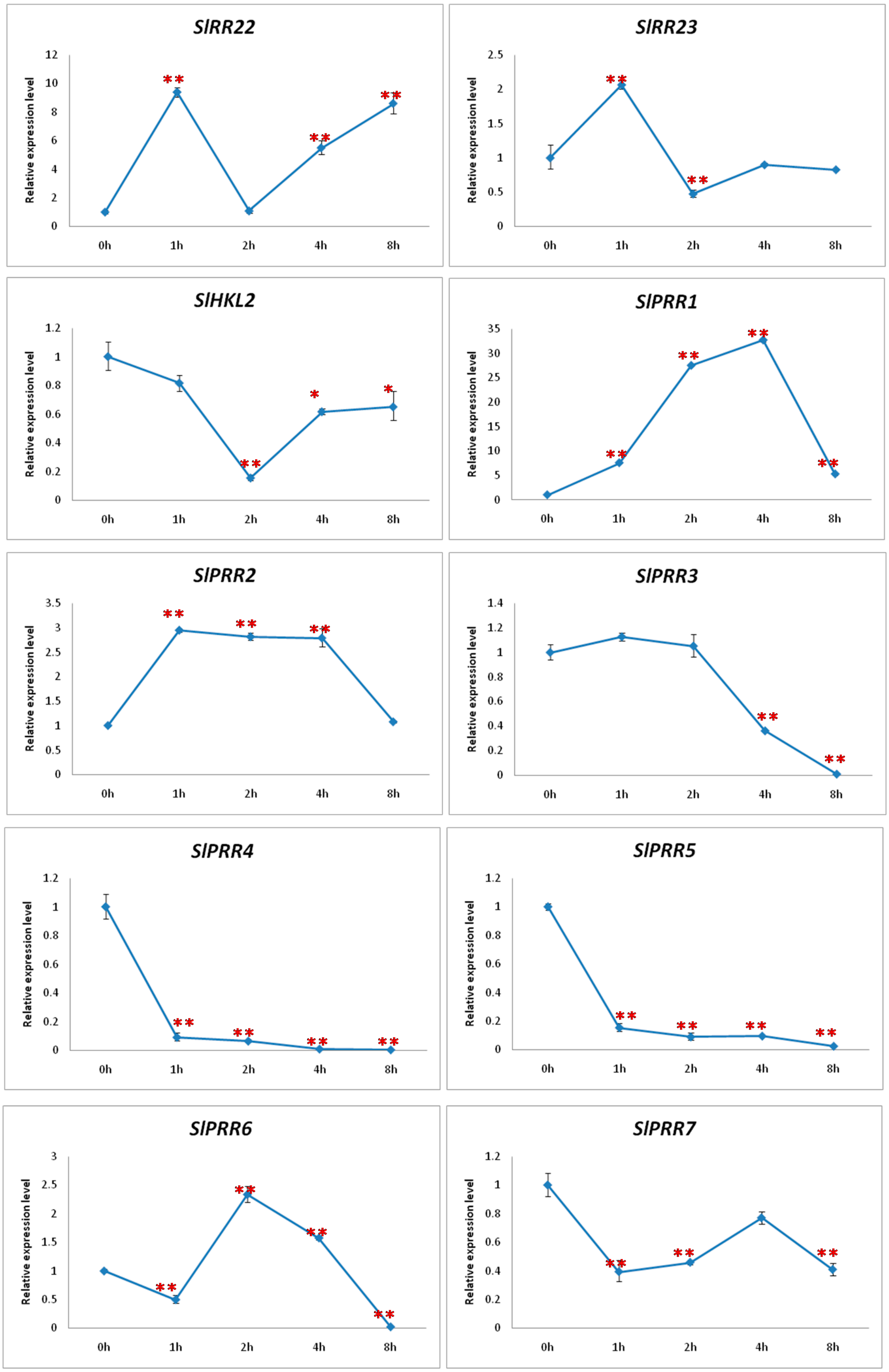
SlRR22
SlRR23
SlHKL2
SlPRR1
SlPRR2
SlPRR3
SlPRR4
SlPRR5
SlPRR6
SlPRR7
Relative expression level
0h
1h
2h
4h
8h

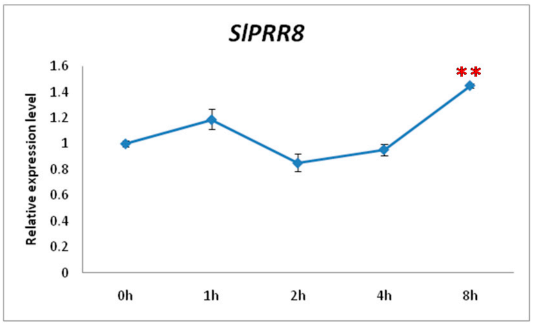

Table S6. Gene-specific primers of selected TCS genes used for subcellular localization and qRT-PCR analysis.

Primers used for subcellular localization

Primer-S	Primer-A	Gene Name
CGGGATCCTGGTGTTGATAAGATAAGAG	GCTCTAGACACACTGTATTCTGATTTC	*SlHK8*
CGGGATCCAAGGAAATGGAAGTGGG	GCTCTAGAATCCCGTAAGCATTGAG	*SlHP2*
CGGGATCCCTTCTCCCCCACATCAAA	GCTCTAGACATGTTTCACCTGCTGC	*SlHP3*
CGGGATCCAGTGAGATGGCAAGAAATG	GCTCTAGAATGGGAGAGGAGGGC	*SlRR1*
CGGGATCCTGGTCTTCAATGGTAAGTATG	GCTCTAGATCATGGGGAACTTGAGACC	*SlRR8*

Primers used for qRT-PCR

Primer-S	Primer-A	Gene Name
ATAACCAACTCCTCTTCGTCC	AAGTCCTTTCGTAACTCAGCC	*SlHK1*
TCAACGGGAGCAAATACTAAC	GATGTCCTCTTCACGCAAAC	*SlHK2*
GGTTAGAGAGAAATGGGGTGC	TATGACAAAAGGTGATGAGGC	*SlHK3*
CTTTGTCTTTGGTGGGGTG	TGCTTACGTTGAACTGGTCC	*SlHK4*
CACCTTCTTTATTCCTCTCATC	CACATCAAACACCTAACCCC	*SlHK5*
TGTCAGGAGAGGAGGATCG	GAATGTCAAAACAACACCAAGG	*SlHK6*
AACGGGGAACACAGACAGAG	CACCAAGAAATCCAAAGACG	*SlHK7*
TATTGGAGATGAAAAACGGC	CAACTTGGGGAAATCCTGAG	*SlHK8*
CTCTGCTACCTATGCCTCC	ATCCCACCATCATCTCCAC	*SlHK9*
GACTTCAAGAAACTGGATGCTC	TTACGGAAGGAAATGCACAC	*SlHP1*
GGTAATCCAAGCAACAAAGC	CCAAGAATCCCTCACGAAAC	*SlHP2*
CGATGAGCATTGATTTGGTA	GAATTTGATGTGGGGGAGA	*SlHP3*
AGATGATGATAACCCTAACTTCG	GAGCACTCTCTTTTCACTCTTTTG	*SlHP4*
CTATGATTTTGCTAGGCTGGA	CATTGTCTTTTTACTCTTCTGGC	*SlPHP1*
CCTCCCCTAATTTCGTCTC	GCCAATGCTTGAACTGC	*SlPHP2*
TGAACAAGAGAAAGTTGCCAG	AGAGGTCAAGTGATGAGGGTG	*SlRR1*
TAGATGTTTGGAGGAAGGTGC	GGGATGGTATTGATTGTGTCG	*SlRR2*
AGCAGCAACAACAACAACAG	TGCGAGCGAGAGATTACAC	*SlRR3*
GATTACTGTATGCCTGGGATG	GCCCCTTCTTCTAAGCATCTAC	*SlRR5*
GCTTAGAAGAAGGGGCAG	TGGGGCTTTTACATTTGG	*SlRR4*
AGATGGTCTGAGGGCTTTAG	GATGACATGATCACAACTGGTAC	*SlRR6*
GAAAATCAAGGAATCGTCG	GTAATTAGCACCCAACACAAC	*SlRR7*
ACCTTCTTGCCTTGTATTGC	CTGGTTTGAGTTCCCTGTC	*SlRR8*
GCATGGTATCGCAAAGGC	GCATCCCACAAACAGCAC	*SlRR9*
GGAGTCACTACAGAACATTTGG	CAACCTCACTTCATTAGTTACACAC	*SlRR13*
GAAGGATCATTCTTTGCTTTCG	GGTATTTCTTTAGACGGTAGGTG	*SlRR16*
TGTAGCTCTATCCATTCTCCG	TCATTTCAATCGTCACCTCTG	*SlRR18*
GTGTTGTTTCAGTGCCAGC	CCAATCCAAAGGAATCGT	*SlRR21*
AGGAGAAGGCGAAAGAGG	AGCCCAAACTACACGAGG	*SlRR22*
GCTCAGGAAATGCCAGTATC	CAAGACCAACAAGTTCAAGAAG	*SlRR23*
